# In vitro and in silico analysis of ‘Taikong blue’ lavender essential oil in LPS-induced HaCaT cells and RAW264.7 murine macrophages

**DOI:** 10.1186/s12906-022-03800-0

**Published:** 2022-12-06

**Authors:** Mengya Wei, Fei Liu, Rifat Nowshin Raka, Jie Xiang, Junsong Xiao, Tingting Han, Fengjiao Guo, Suzhen Yang, Hua Wu

**Affiliations:** 1 Beijing Technology and Business University, Beijing, 100048 China; 2 Shandong Freda Biotech Co., Ltd, Ji’nan, 250101 Shandong China; 3 Xinjiang Eprhan Spices Co., Ltd, Cocodala, 835213 Xinjiang China

**Keywords:** Lavender essential oil, Skin, Anti-inflammatory, Antioxidative, Macrophages

## Abstract

**Background:**

‘Taikong blue’ lavender, a space-bred cultivar of *Lavandula angustifolia,* is one of the main lavender essential oil production crops in Xinjiang Province, China. Several cases of local usage indicated that ‘Taikong blue’ lavender essential oil (TLEO) had excellent anti-inflammatory and antioxidant properties for skin problems. However, to date, substantial data on these functions are lacking. In this study, we aimed to investigate the composition and bioactivities of TLEO and the potential underlying mechanisms through LPS-induced inflammatory models of HaCaT and RAW264.7 cells.

**Methods:**

The composition of TLEO was determined by GC‒MS. To study the anti-inflammatory and antioxidative properties of TLEO, we induced HaCaT and RAW264.7 cells by LPS. TLEO (0.001%-0.1%, v/v) was used to treat inflamed cells with dexamethasone (DEX, 10 μg/mL) as the standard drug. A variety of tests were carried out, including biochemical assays, ELISA, RT‒PCR, and western blotting. Docking of components was performed to predict potential ligands.

**Results:**

The GC‒MS analysis revealed that 53 compounds (> 0.01%) represented 99.76% of the TLEO, and the majority of them were esters. TLEO not only reduced the levels of oxidative stress indicators (NO, ROS, MDA, and iNOS at the mRNA and protein levels) but also protected the SOD and CAT activities. According to the RT‒PCR, ELISA, and Western blot results, TLEO decreased inflammation by inhibiting the expression of TNF-α, IL-1β, IL-6, and key proteins (IκBα, NF-кB p65, p50, JNK, and p38 MAPK) in MAPK-NF-кB signaling. Molecular docking results showed that all of the components (> 1% in TLEO) were potent candidate ligands for further research.

**Conclusion:**

The theoretical evidence for TLEO in this study supported its use in skin care as a functional ingredient for cosmetics and pharmaceutics.

**Graphical Abstract:**

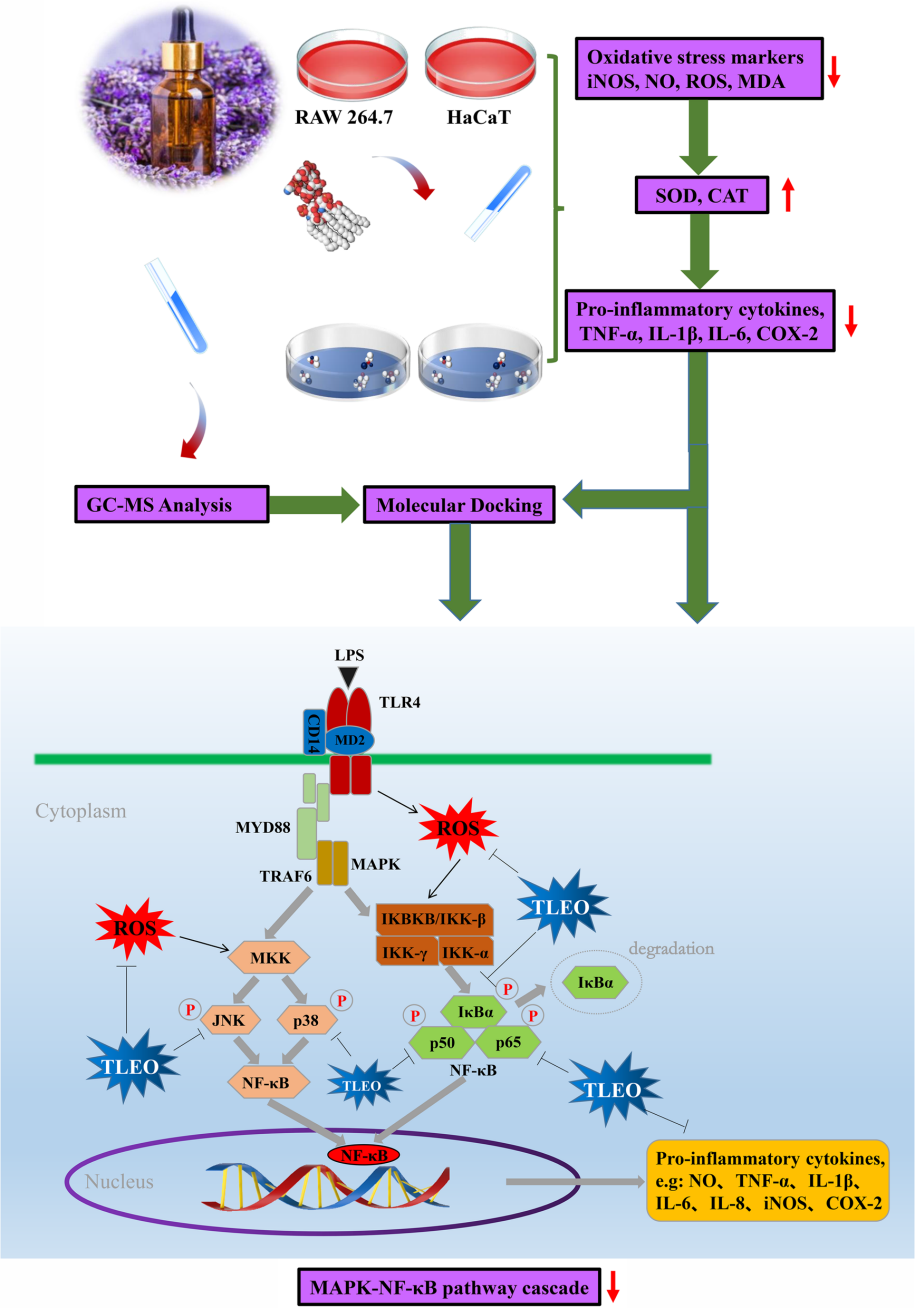

**Supplementary Information:**

The online version contains supplementary material available at 10.1186/s12906-022-03800-0.

## Introduction

Skin is the largest organ of the body as well as the first defensive line to protect the organism against external stimulation, while macrophages are immune cells that protect host homeostasis by phagocytosis, cytokine production, and antigen presentation [1, 2]. However, skin inflammation presents in common apparent forms, such as erythema, edema, mossy skin, and scaly plaques. It afterward results in other symptoms, such as severe itching, skin aging, lesions, insomnia, mental anxiety, etc. [3–6]. Inflammation plays a crucial role in different types of skin problems and diseases. Excessive inflammation promotes the overproduction of proinflammatory mediators and imbalances the immune system. This process involves the regulation of several enzymatic systems and signaling pathways, such as MAPK, TLR4, JAK-STAT, and NF-кB. While NO, ROS and iNOS are major and typical markers of oxidative stress, TNF-α, IL-1β, and IL-6 are the most common proinflammatory cytokines. As a consequent response to inflammation, IκBα, NF-кB p65 and p50, JNK, and p38 MAPK are phosphorylated and eventually enhance the MAPK-NF-κB pathway [7].

Therefore, managing uncontrolled inflammation could be a good way to regulate inflammatory responses. Currently, there are two types of anti-inflammatory drugs used in therapeutics: steroidal anti-inflammatory drugs (SAIDs) and nonsteroidal anti-inflammatory drugs (NSAIDs) [8]. Nevertheless, some of these drugs always have certain side effects and random adverse reactions, including dry skin, desquamation, skin atrophy, telangiectasia, and erythema [9]. Therefore, natural plant extracts without side effects have received extensive attention due to their safety and effectiveness.

Lavender essential oils (LEOs) are pale-yellow liquids with intense floral-herbal lavender scents and are mainly obtained by steam or hydro distillation from aerial parts of blooming lavenders [10]. LEOs have shown various bioactivities, including antidepressive, antioxidative, anti-inflammatory, antiplatelet, antithrombotic, antimutagenic, carminative (smooth muscle relaxing), and sedative activities, in different studies. These treatments are also effective for wounds, burns, insect bites, urinary infections, cardiac diseases, eczema, and even blood sugar reduction [11, 12]. *Lavandula angustifolia* Mill was brought to Xinjiang, China, in the 1950s. 'Taikong blue' lavender is a new variety of *L. angustifolia* Mill produced by mutagenesis in space breeding techniques. From September 27th to October 15th, 2004, 100 g *L. angustifolia* Mill seeds were released into space on a Long March 2-D carrier rocket as the 20th recoverable science and technology experimental satellite. After a year of cultivation, the researchers discovered that the first space seeds of 'Taikong blue' lavender had numerous benefits over *L. angustifolia* Mill cultured in the same place, such as longer blooming periods and higher oil yield, and it has become a significant cultivated species in Xinjiang [13, 14].

Our preliminary experimental verification demonstrates that 'Taikong blue' lavender essential oil (TLEO) significantly inhibited LPS-induced IL-6 gene expression (*p* < 0.05) and was even better than two other lavender essential oils from Xinjiang: 'French blue' lavender essential oil (FLEO) and 701 lavender essential oil (701 LEO) (see supplementary data Fig. S8), indicating that TLEO may have good anti-inflammatory activity. However, its anti-inflammatory and antioxidative activity and related mechanisms are less studied.

Keratinocytes constitute 95% of the skin's outer layer, and as the primary interface, they not only provide barrier protection but also participate in the original immune modulatory responses. With the development of inflammatory reactions, macrophages in the inner skin play a vital role as inflammatory controllers to monitor and secrete various stimulators and cytokines to participate in immune regulation. In other words, macrophage cells protect the skin hemostasis while keratinocytes provide immunological responses [15]. Therefore, in this study, we investigated TLEO’s chemical composition and its *in vitro* anti-inflammatory and antioxidative potential in an LPS-induced inflammatory model of human keratinocytes (HaCaT) and RAW264.7 murine macrophages. In addition, we used molecular docking to determine the potential interactions between TLEO components and pathway proteins.

## Materials and methods

### Chemical reagents and antibodies

TLEO ≥ 98% (HPLC) was purchased from Xinjing Eprhan Spices Co., Ltd. Lipopolysaccharides (LPSs, from *Escherichia coli*) and dexamethasone (DEX) were purchased from Sigma (St. Louis, USA). Dulbecco’s modified Eagle’s medium (DMEM) and phosphate buffered solution (PBS) were purchased from HyClone (USA), and 3-(4,5-dimethylthiazol-2-yl)-2,5-diphenyltetrazolium (MTT) was purchased from Beyotime Biotechnology Co., Ltd. (Shanghai). Fetal bovine serum (FBS), glutamine, penicillin/streptomycin (P/S), and 0.25% trypsin were obtained from Zhongsheng Aobang Biotechnology Co., Ltd. (Beijing). ROS, NO, MDA, SOD, and CAT assay kits were purchased from Beyotime Biotechnology Co., Ltd. (Shanghai). Antibodies for Western blot and ELISA kits were from ABclonal Biotechnology Co., Ltd. (Wuhan).

### GC‒MS Analysis

GC–MS (Thermo Fisher Scientific, Waltham, MA) with a Thermo Fisher Trace 1310 GC system, a Thermo Fisher ISQ LT mass detector, and a DB-FFAP (30 m × 0.25 mm × 0.25 μm; J & W Scientific, Folsom, CA, USA) column was used for the TLEO analysis. One microliter of PREO and 1 μL of 2-nonyl ketone were dissolved in 998 μL of methylene chloride, mixed well, and filtered with a 0.22 μm filter. The mass-selective detector was operated in electron-impact ionization (EI) mode with a mass scan range from m/z 50 to m/z 500 at 70 eV. The oven initial temperature was maintained at 40 °C for 3 min, raised to 50 °C at a rate of 6 °C/min, 7 °C/min to 130 ℃, and then held for 1 min, 2 °C/min to 140 ℃ for 1 min, 3 °C/min to 150 ℃ for 1 min, 5 °C/min to 160 ℃ for 1 min, and finally 230 °C at a rate of 7 °C/min for 5 min. The injection temperature and detection temperature were 250 °C, and there was no split. Helium was the carrier gas at a flow rate of 1.3 mL/min. The TLEO constituents were identified by comparing the mass fragmentation profiles and the retention indices of the chromatographic peaks with the National Institute of Standards and Technology (NIST) MS spectral database (version 2017).

### Cell lines, growth conditions, and treatments

HaCaT, a spontaneously immortalized human keratinocyte cell line, was purchased from the Peking Union Cell Resource Center (Beijing, China). HaCaT cells were cultured in DMEM with 10% (v/v) FBS, 1% (v/v) P/S, and 1% (v/v) glutamine. RAW264.7 murine macrophages were purchased from Shanghai Fuheng Biological Co., Ltd., and cultured in DMEM with 10% FBS, 1% P/S, 1% sodium pyruvate, and 1% glutamine. The cells were maintained in a humidified 5% CO_2_ incubator at 37 °C and were subcultured every 2–3 days to maintain logarithmic growth. After 24 h of culture, HaCaT and RAW264.7 cells were treated with LPS (2.5 μg/mL for 20 h and l μg/mL for 12 h, respectively). Later, the DEX solution (10 μg/mL) or different concentrations (0.001, 0.01, 0.1%) of TLEO containing serum-free media were added to the cell cultures. After 12 h, samples were collected and processed for the following experiments.

### Cell viability assay

The cytotoxic activities of TLEO alone were measured by colorimetric MTT [3-(4,5-dimethyl-2-thiazolyl)-2,5-diphenyltetrazolium bromide] assays with HaCaT and RAW264.7 cells. HaCaT and RAW264.7 cell suspensions were added to each well of a 96-well plate, and the cell number in each well was approximately 2 × 10^3^. After 24 h of incubation, different concentrations (0.001–1% v/v) of TLEO were added to each well and cultured for 12 h. Then, the solution was carefully removed and added to 10 µL of 5 mg/mL MTT. After incubation at 37 °C for 4 h, the resulting formazan crystals were dissolved in 100 µL of sodium dodecyl sulfate solution (Macklin, Shanghai, China). The absorbance was measured using the microplate reader Infinite M200PRO (Tecan) at a wavelength of 550 nm.

For analysis of the roles of TLEO in LPS-induced protective effects, HaCaT and RAW264.7 cells were grown and treated in 96-well plates as described above. After 12 h of culture, the cells were processed for MTT assays as described previously [15]. Cell cytotoxicity was calculated as the percentage of MTT absorption by using the following formula:

$$\mathrm{Cell}\;\mathrm{viability}\;\left(\%\right)\;=\;\left({\mathrm{OD}}_{\mathrm{sample}}-{\mathrm{OD}}_{\mathrm{blank}}\;\right)/{\mathrm{OD}}_{\mathrm{control}}-{\mathrm{OD}}_{\mathrm{blank}})\times100$$

### Reactive Oxygen Species (ROS) level determination and Nitric Oxide (NO) production assay

HaCaT and RAW264.7 cell suspensions were added to each well of a 96-well plate. Finally, the cell number in each well was approximately 2 × 10^3^ to determine ROS and NO levels. ROS production was identified using the ROS Detection Kit (Beyotime Biotechnology). In brief, the supernatant was discarded, and 2,7-dichlorodihydrofluorescein diacetate (DCFH-DA) (10 μM/L; Beyotime Biotechnology) diluted with DMEM was added to the plate. The cells were then cultured for 20 min in the incubator. Cells were washed three times using DMEM without FBS to remove DCFH-DA from the plate. The ROS level was measured using a microplate reader infinite with excitation at 488 nm and emission at 525 nm.

After the treatments described above, the NO production assay was performed in triplicate by using the Griess assay according to the instructions given by the kit. The absorbance values of the colored solution were measured using a microplate reader at 540 nm. These values were converted to micromoles per liter (μmol/L) with a standard curve obtained by adding 0–80 μmol/L sodium nitrate to fresh culture media. This experiment was performed in triplicate individually [16].

### Biochemical assay

Three milliliters of HaCaT and RAW264.7 cells at the logarithmic growth stage were inoculated in 35 mm dishes at a density of 1.5 × 10^6^ cells/dish and 1.8 × 10^6^ cells/dish, respectively. After the treatments described in 2.3, measurement of MDA levels in cells was performed using a Lipid Peroxidation MDA Assay Kit. MDA is an end-product of fatty acid peroxidation and can be measured via a chromogenic reaction between MDA and thiobarbituric acid (TBA). Briefly, 100 μL of cell lysate was mixed with 200 μL of MDA working solution followed by incubation in a 100 °C water bath for 15 min and then cooled to 25 °C. The mixture was centrifuged at 1000 × g for 10 min, and 200 μL of supernatant was used to measure absorbance at 532 nm. The TBARS concentration was calculated from an MDA standard curve.

For the CAT activity assay, cells were treated as described in 2.3. The cell lysates were centrifuged at 1600 rpm at 4 °C for 20 min, the supernatant was diluted to the proper concentration, and the protein concentrations were determined by a BCA protein assay kit (Beyotime Institute of Biotechnology). The catalase activity was measured following the kit’s instructions by a microplate reader at a wavelength of 520 nm.

SOD activity was measured by a total superoxide dismutase assay kit with WST-8. After the same treatments described in 2.3, the protein concentrations of the cell lysate supernatant were determined by a BCA protein assay kit (Beyotime Biotechnology), and SOD activity was measured by an infinite microplate reader at a wavelength of 450 nm according to the manufacturer’s instructions [15, 16].

### qRT‒PCR

Quantitative real-time polymerase chain reaction (qRT‒PCR) was used to detect the mRNA levels of proinflammatory cytokines. Three milliliters of cells were plated in a petri dish (35 mm) at a density of 1.5 × 10^6^ cells/dish (HaCaT) and 1.8 × 10^6^ cells/dish (RAW264.7 cells). Briefly, after the treatments of the cells as described in 2.3, total RNA was extracted from the cells with TRIzol. Complementary DNA (cDNA) synthesis was conducted with a ReverTra Ace qPCR RT kit. Quantitative PCR was performed on a CFX96™ real-time PCR machine. The mRNA level was calculated using the 2^−∆∆CT^ method [17]. The sequences of primers for qRT‒PCR are presented in Table 1.

**Table 1:** Primers for detection of gene expression through qRT-PCR

Genes	Forward	Reverse
β-actin	CCTAGAAGCATTTGCGGTGCACGATG	TCATGAAGTGTGACGTTGACATCCGT
TNF-α	TACAGGCTTGTCACTCGAATT	ATGAGCACAGAAAGCATGATC
IL-1β	TGCAGAGTTCCCCAACTGGTACATC	GTGCTGCCTAATGTCCCCTTGAATC
IL-6	AAGTGCATCATCATCGTTGTTCATACA	GAGGATACCACTCCCAACAGACC

### Enzyme-linked immunosorbent assay (ELISA)

HaCaT cells **(**1.5 × 10^6^ cells/dish) and RAW264.7 cells (1.8 × 10^6^ cells/dish) were inoculated in 35 mm dishes and incubated for 24 h at 37 °C in 5% CO_2_. After the same treatments of the cells as described in 2.3, a brief centrifugation step at 2000 × g was applied to remove dead cells and cell debris, and the supernatants were subjected to ELISAs. ELISAs were performed following the manufacturer’s instructions for TNF-α, IL-1β, and IL-6 levels.

### Western blot (WB)

A total of 1.5 × 10^6^ cells/dish HaCaT cells and 1.8 × 10^6^ cells/dish RAW264.7 cells (each 3 mL) were seeded in dishes (35 mm). The cells were treated in the same way as in 2.3. Then, the cells were washed with cold PBS three times, and the proteins were extracted with a Total Protein Extraction kit. Protein concentrations were determined by a BCA protein assay kit. Equal amounts of proteins were analyzed in polyacrylamide gels by SDS‒PAGE and subsequently transferred to PVDF membranes. PVDF membranes were blocked with 5% nonfat milk in PBST and incubated with primary antibody overnight at 4 °C. Subsequently, the PVDF membranes were washed with PBST and incubated with secondary antibody for 1 h at room temperature. Bands were analyzed by chemiluminescence (Tanon 5200, Beijing Yuanping Hao Biotech) with horseradish peroxidase-conjugated IgG [15].

### Molecular docking analysis

The ADME properties of all TLEO components were predicted using Swiss-ADME. The 3D conformations of all 11 components (< 1% from GC‒MS result) were downloaded from the PubChem database (see Supplementary Fig. S1). The ligands were prepared by energy minimization using the MMFF94 forcefield. After analysis of the sequence, the 3D structures of the IKB-α (Human (H):1NFI; Mouse (M): SWISS-MODEL), JNK (H:3NPC; M: SWISS-MODEL), p50 (H:2O61_B chain; M:2V2T), p65 (H: 2O61_A chain; M: 6GGR) and p38MAPK (H:1M7Q; M:6SOI) proteins for humans and mice were obtained from PDB or constructed when needed [18, 19] (see Supplementary Table S2 and Fig. S2). The proteins were energy minimized by Swiss-PDB and were prepared by cleaning and adding charges. Molecular docking was performed using the CBDOCK web server, and the best model with the lowest RMSD was chosen for each protein-component docking [20]. Discovery Visual Studio (DSV) software was used to study the interaction of docked complexes.

### Statistical analysis

All assays were conducted at least three times with three different sample preparations. All data are expressed as the means ± standard deviations (SD). Analysis of variance (ANOVA) was performed using SPSS software (version 19.0; SPSS, Inc., USA). One-way ANOVA and Scheffe’s method were used to analyze the differences between the means. A significant difference was recognized at the level of *p* < 0.05. The figures in this paper were drawn by Origin 2019b.

## Results

### Chemical profiling of TLEO

For TLEO, 53 compounds were identified and are listed in Table 2. Six major groups of compounds, which resulted in 99.73% yield of TLEO, were detected: esters (51.08%), alcohols (33.14%), monoterpenoids (7.11%), sesquiterpenoids (4.74%), monoterpenes (3.35%), and ketones (0.09%). The remaining 0.22% was composed of cryptone and p-cymen-8-ol. The total ion chromatogram was presented as Fig. S9. (Please see supplementary data).

**Table 2:** GC–MS analysis to determine the composition of TLEO

Serial	Compounds	RI	Base Peak (M^+^)		Mass Fragmentation	Relative Peak Area (%)
*Monoterpenoids*						
1	α-Pinene	1028		136	93, 92, 91, 77, 79	0.21
	Camphene	1071		136	93, 121, 79, 91, 39	0.08
	β-Pinene	1112		136	93, 41, 69, 39, 91	0.05
4	Sabinene	1124		136	93, 91, 77, 79, 41	0.04
5	3-Carene	1147		136	93, 91, 79, 77, 92	0.10
6	D-Limonene	1200		136	68, 93, 67, 79, 94	0.48
7	Eucalyptol	1213		154	43, 81, 93, 71, 69	0.95
8	**cis-β-Ocimene**	1235		**136**	**93, 91, 79, 80, 77**	**3.04**
9	**β-Ocimene**	1250		**136**	**93, 91, 79, 80, 77**	**1.20**
10	Terpinolene	1283		136	93, 121, 91, 79, 77	0.09
11	Linalool oxide	1445		170	59, 94, 43, 93, 111	0.09
12	Trans-linalool oxide	1452		170	59, 94, 43, 55, 68	0.08
13	Camphor	1518		152	95, 81, 108, 69, 41	0.16
14	Borneol	1675		154	95, 110, 41, 67, 55	0.49
15	Carvone	1740		150	82, 54, 39, 93, 108	0.05
*Monoterpenes*						
16	**β-Myrcene**	1161		**136**	**93, 69, 41, 91, 79**	**1.10**
17	α-Terpine	1180		136	121, 93, 91, 77, 79	0.04
18	**(4E,6E)-Alloocimene**	1379		**136**	**121, 79, 105, 91, 77**	**1.23**
19	Bornyl acetate	1581		196	95, 121, 93, 43, 136	0.15
20	Lavandulol	1677		154	69, 41, 111, 68, 93	0.37
21	Cuminal	1788		148	133, 105, 77, 79, 91	0.10
22	Geraniol	1847		154	69, 41, 68, 29, 93	0.36
*Sesquiterpenoids*						
23	α-Copaene	1492		204	161, 119, 105, 91, 93	0.07
24	α-Santalene	1576		204	94, 93, 41, 91, 95	0.25
25	β-Copaene	1586		204	161, 105, 91, 119, 120	0.02
26	**Caryophyllene**	1595		**204**	**93, 133, 91, 41, 79**	**2.49**
27	cis-β-Farnesene	1662		204	41, 69, 93, 67, 133	0.04
28	trans-β-Farnesene	1664		204	41, 69, 93, 67, 79	0.99
29	Humulene	1667		204	93, 41, 80, 121, 91	0.08
30	D-Germacrene	1710		204	161, 105, 91, 119, 81	0.42
31	Caryophyllene oxide	1989		220	43, 41, 79, 93, 69	0.16
32	T-Cadinol	2169		222	161, 204, 105, 81, 43	0.22
*Alcohols*						
33	1-Octen-3-ol	1450		128	57, 72, 43, 55, 41	0.43
34	**Linalool**	1547		**154**	**71, 93, 55, 43, 41**	**29.48**
35	**Terpinen-4-ol**	1602		**154**	**71, 111, 43, 93, 41**	**2.44**
36	α-Terpineol	1697		154	59, 93, 136, 121, 43	0.64
37	Nerol	1797		154	69, 41, 93, 68, 67	0.12
38	Cuminol	2113		150	135, 105, 79, 107, 91	0.03
*Esters*						
39	Butyl acetate	1074		116	43, 56, 41, 61, 73	0.04
40	Butyl butylate	1220		144	71, 89, 56, 43, 41	0.14
41	Hexyl acetate	1272		144	43, 56, 55, 61, 41	0.56
42	Hexyl isobutyrate	1339		172	43, 89, 71, 56, 84	0.02
43	Hexyl formate	1352		130	56, 55, 41, 43, 42	0.02
44	**1-Octen-3-yl acetate**	1380		**170**	**43, 99, 54, 41, 67**	**2.62**
45	Hexyl butanoate	1414		172	43, 71, 89, 56, 41	0.33
46	**Linalyl acetate**	1555		**196**	**93, 43, 41, 80, 69**	**40.97**
47	**Lavandulol acetate**	1606		**196**	**69, 93, 43, 41, 68**	**4.77**
48	Nerol acetate	1724		196	69, 41, 43, 68, 93	0.50
49	**Geranyl acetate**	1752		**196**	**69, 41, 43, 68, 93**	**1.11**
*Ketones*						
50	3-Octanone	1253		128	43, 57, 99, 72, 71	0.04
51	Coumarin	2454		146	118, 89, 90, 63, 62	0.05
*Others*						
52	Cryptone	1687		138	96, 95, 67, 41, 43	0.18
53	p-Cymen-8-ol	1852		150	43, 135, 91, 65, 136	0.04
Monoterpenes						3.35
Sesquiterpenoids						4.74
Alcohols						33.14
Esters						51.08
Ketones						0.09
Others						0.22
Totals						99.73

^a^ Retention index relative to n-alkanes on DB-WAX column

^b^ Relative percentage based on peak area. Components with bold font were chosen as the ligand for docking. Bold fonts indicate the components(> 1%) used as ligands in this study

### Effects of TLEO on LPS-treated cell viability

**Fig. 1:**
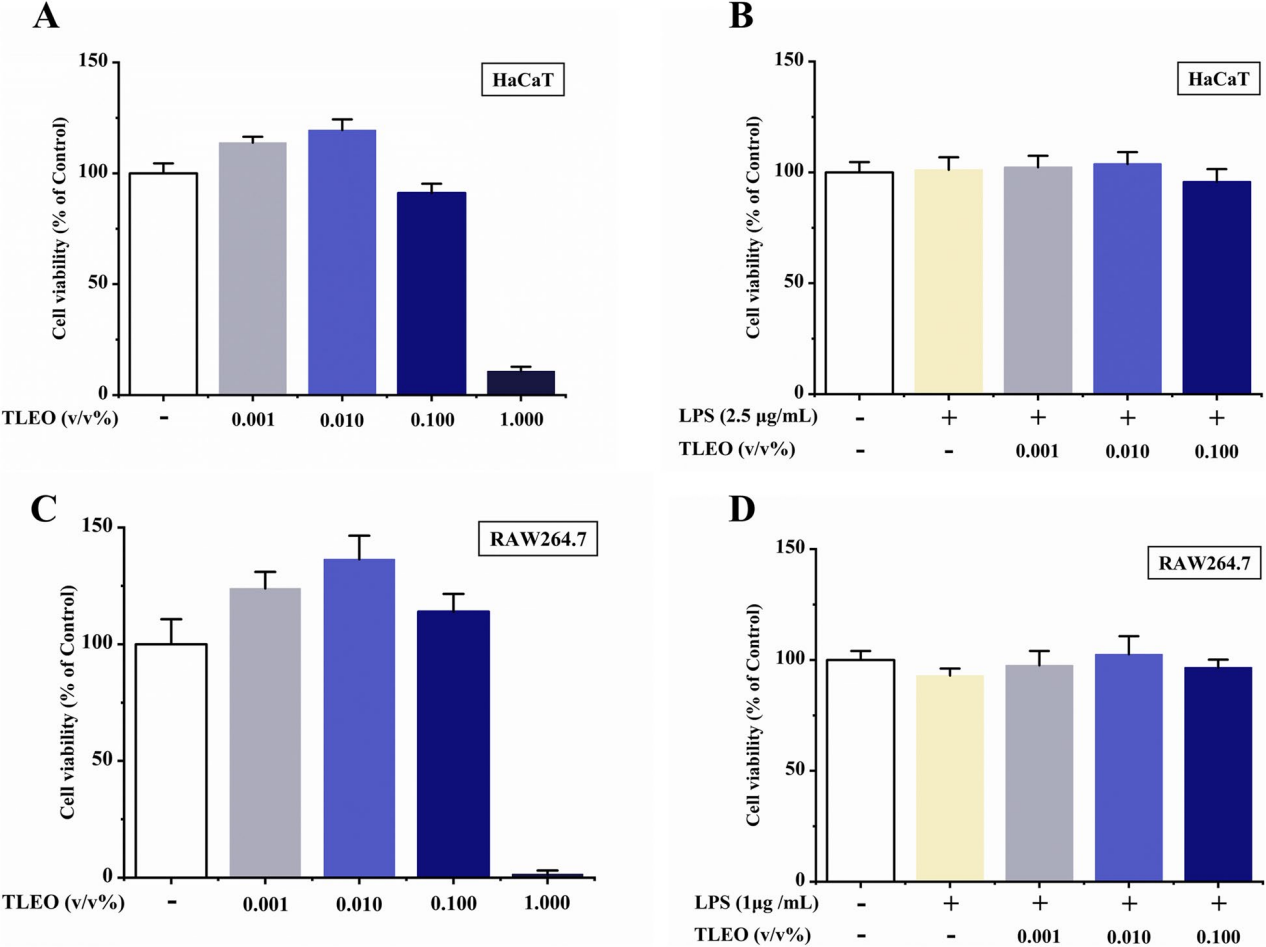
Cell viabilities of TLEO using MTT assay in HaCaT cells and RAW264.7 murine macrophages. **A:** Effects of TLEO on HaCaT cell cytotoxicity (without LPS); **B:** Effects of TLEO on LPS-induced HaCaT cells; **C:** Effects of TLEO on RAW264.7 cell cytotoxicity (without LPS); **D:** Effects of TLEO on LPS induced RAW264.7 cells. Data are mean ± S.D. (*n* = 6). Statistical analysis was performed by one-way ANOVA with a scheffe’s test

Different doses of TLEO were added to cell cultures with or without LPS treatments to determine their effects on cell viability. As shown in Fig. 1A-D, the cell viabilities of both cell lines were more than 90% with TLEO (0.001–0.1%) and less than 25% under 1% TLEO treatment. The cell viabilities of subsequent experiments were all over 95% and did not show any decreases when 0.001–0.1% TLEO was administered to the two LPS-induced inflammatory cell models. Therefore, TLEO concentrations of 0.001–0.1% were safe and used for the subsequent experiments.

### Effects of TLEO on oxidative stress responses in the LPS-induced inflammatory cell model

**Fig. 2:**
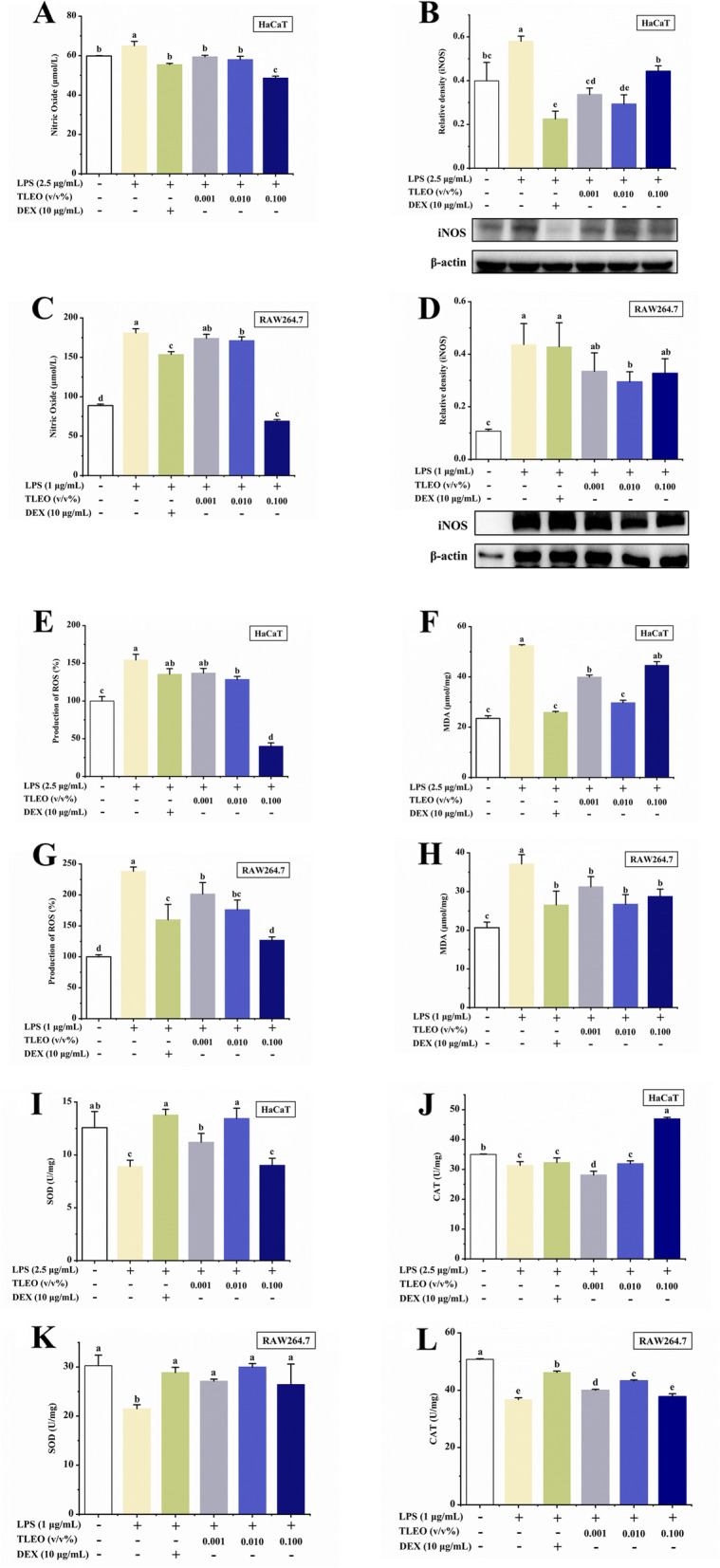
Effects of TLEO on the oxidative stress markers in LPS-induced HaCaT cells and RAW264.7 murine macrophages. **A:** Effects of TLEO on NO production in LPS-induced HaCaT cells; **B:** Effects of TLEO on iNOS protein expression in LPS-induced HaCaT cells; **C:** Effects of TLEO on NO production in LPS-induced RAW264.7 cells; **D:** Effects of TLEO on iNOS protein expression in LPS-induced RAW264.7 cells; **E, F:** Effects of TLEO on ROS production and MDA activity in LPS-induced HaCaT cells; **G, H:** Effects of TLEO on ROS production and MDA activity in LPS induced RAW264.7 cells; **I.J:** Effects of TLEO on SOD and CAT activities in LPS-induced HaCaT cells; **K, L:** Effects of TLEO on SOD and CAT activities in LPS-induced RAW264.7 cells; The data are the means ± S.D. (n = 3). Statistical analysis was performed by one-way ANOVA with a scheffe’s test. “a”; “b”; “c”; “d” indicates a significant difference (*p* < 0.05) compared with the LPS-treated group

As shown in Fig. 2, almost all the TLEO treatments successfully and significantly (*p* < 0.05) alleviated inflammatory mediator and oxidative factor production and protected antioxidative enzyme activities, similar to the control or even better. Notably, macrophages are more sensitive to LPS treatment. TLEO (0.1%) inhibited NO and ROS production to a lesser extent than DEX treatment. Compared with LPS-treated HaCaT and RAW264.7, the NO yield in 0.1% TLEO treatment was reduced by about 16.36 μmol/L and 111.83 μmol/L, respectively, which was lower than the NO reduction after DEX treatment (9.54 μmol/L and 27.37 μmol/L respectively). However, for the inhibition of iNOS protein expression, the best results were found with 0.01% TLEO (*p* < 0.05) in the two cell lines. The inhibition rates of iNOS in the 0.01% TLEO treatment group were approximately 49.49% and 32.49%. 0.1% TLEO reduced the ROS production of HaCaT and RAW264.7 stimulated by LPS, 74.09%, and 46.75%, respectively, which were higher than those in the DEX treatment group (12.22% and 32.80%). While for the inhibition of MDA production, 0.01% TLEO was used (*p* < 0.05) in HaCaT cells, which reduced the LPS-induced MDA of 52.42 μmol/mg to 29.70 μmol/mg. And 0.001–0.1% TLEO resulted in the same significance (*p* < 0.05) downregulation in the RAW264.7 cell line, the content of MDA was reduced by 5.88 μmol/mg, respectively, 10.38 μmol/mg and 8.37 μmol/mg. The SOD activity of the two LPS-stimulated cell lines under 0.01% TLEO treatment recovered to 13.45 U/mg and 30.00 U/mg, respectively, and the effect was similar to that of the DEX treatment group (13.77 U/mg and 28.88 U/mg), and both reached the normal level (12.58 U/mg and 30.27 U/mg). The same phenomenon was found with CAT only under treatment with 0.1% TLEO in LPS-treated HaCaT cells (*p* < 0.05), 0.1% TLEO increased CAT activity by 15.58 U/mg.

### Effects of TLEO on the expression of inflammatory cytokines

**Fig. 3:**
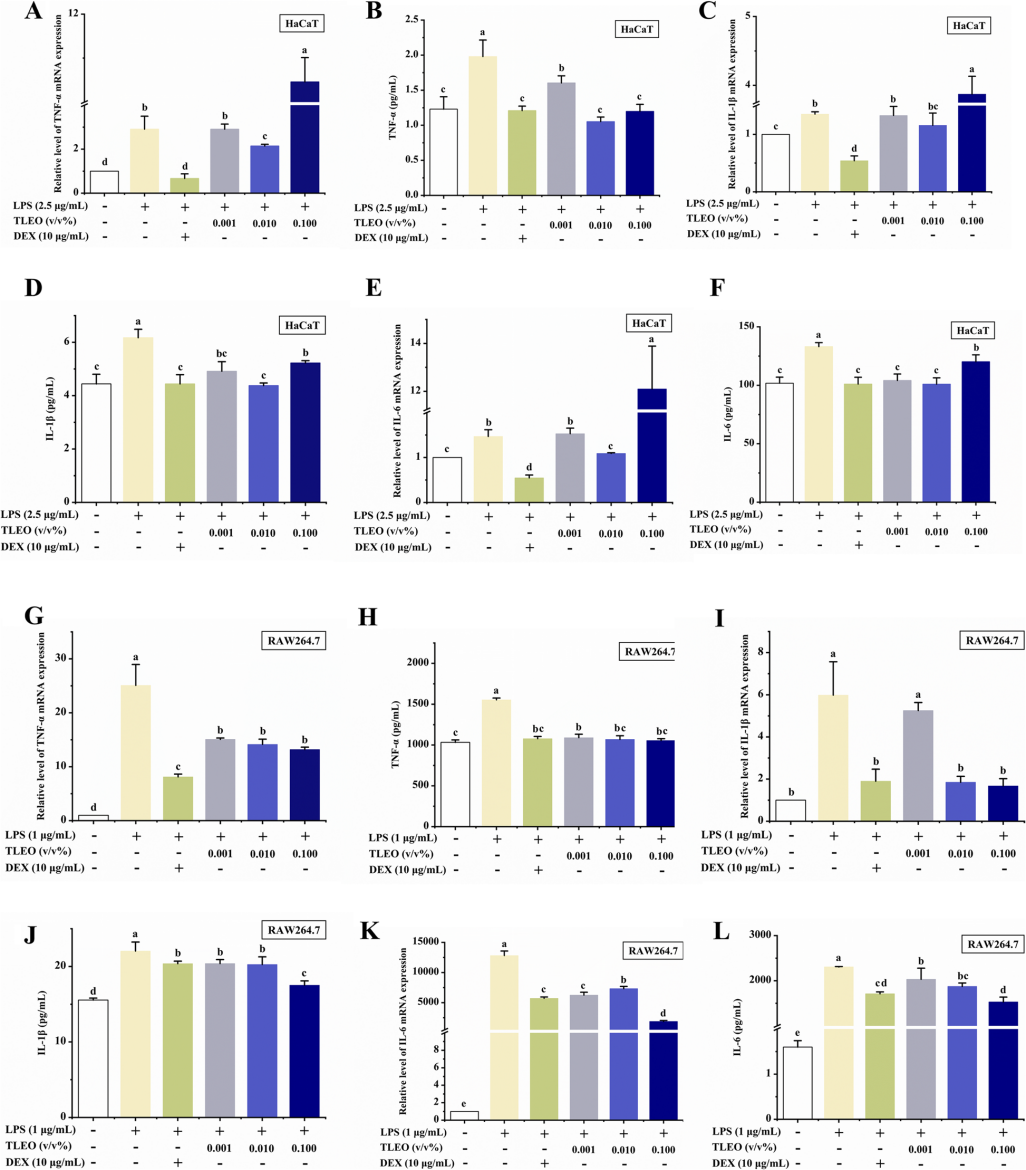
Effects of TLEO on mRNA and protein level of pro-inflammatory cytokines by LPS-induced HaCaTand RAW264.7 cells. **A, B:** Effects of TLEO on the expression of TNF-α at mRNA and protein level in LPS-induced HaCaT cells; **C, D:** Effects of TLEO on the expression of IL-1β at mRNA and protein level in LPS-induced HaCaT cells; **E, F:** Effects of TLEO on the expression of IL-6 at mRNA and protein level in LPS induced HaCaT cells; **G, H:** Effects of TLEO on the expression of TNF-α at mRNA and protein level in LPS induced RAW264.7 cells; **I, J:** Effects of TLEO on the expression of IL-1β at mRNA and protein level in LPS induced RAW264.7 cells; **K, L:** Effects of TLEO on the expression of IL-6 at mRNA and protein level in LPS induced RAW264.7 cells. The data are the means ± S.D.(n = 3). Statistical analysis was performed by one-way ANOVA with a scheffe’s test. “a”; “b”; “c”; “d” indicates a significant difference (*p* < 0.05) compared with the LPS-treated group

TLEO inhibited the gene expression of TNF-α, IL-1β, and IL-6 (*p* < 0.05) at the mRNA and protein levels in the LPS-treated RAW264.7 cells. At the mRNA expression level, TLEO inhibition rates for TNF-α, IL-1β, and IL-6 ranged from 40 to 50%, 12%-72%, and 40%-85%, respectively. While at the protein level, 0.001%-0.1% TLEO reduced the production of TNF-α treated with LPS by 462.79 pg/mL, 484.50 pg/mL, and 497.73 pg/mL, respectively, which was similar to that of the DEX treatment group (476.11 pg/mL). 0.001%-0.1% TLEO reduced the production of IL-1β in LPS treatment to 20.39 pg/mL, 20.26 pg/mL, and 17.52 pg/mL, respectively, similar to the DEX treatment group (20.37 pg/mL). 0.001%-0.1% TLEO reduced the production of IL-6 treated with LPS by 272.87 pg/mL, 432.60 pg/mL, and 774.83 pg/mL, respectively, with certain dose dependence. However, in the case of LPS-activated HaCaT cells, DEX showed the best inhibition of cytokine expression, the inhibition rates of TNF-α, IL-1β, and IL-6 reached 76.98%, 60.03%, and 62.65%, respectively (Fig. 3). The production of TNF-α, IL-1β, and IL-6 was increased by only LPS, while the addition of 0.1% TLEO with LPS further promoted it. The increased expression of the cytokines was reduced significantly by DEX and slightly by 0.01% TLEO. The inhibition rates of 0.01% TLEO on mRNA expression of TNF-α, IL-1β, and IL-6 were 26.34%, 14.51%, and 25.81%, respectively. Similarly, the 0.01% TLEO treatment group achieved the best inhibitory effect on the secretion of these three inflammatory factors. However, in the LPS-treated RAW264.7 cells, TLEO exhibited a dose-dependent effect on the proliferation and LPS-induced expression of TNF-α, IL-1β, and IL-6 mRNA and protein. On average, 0.1% TLEO resulted in comparatively better results than DEX and other TLEOs (Fig. 3) (*p* < 0.05).

### Effects of TLEO on NF-κB and MAPK activation

**Fig. 4:**
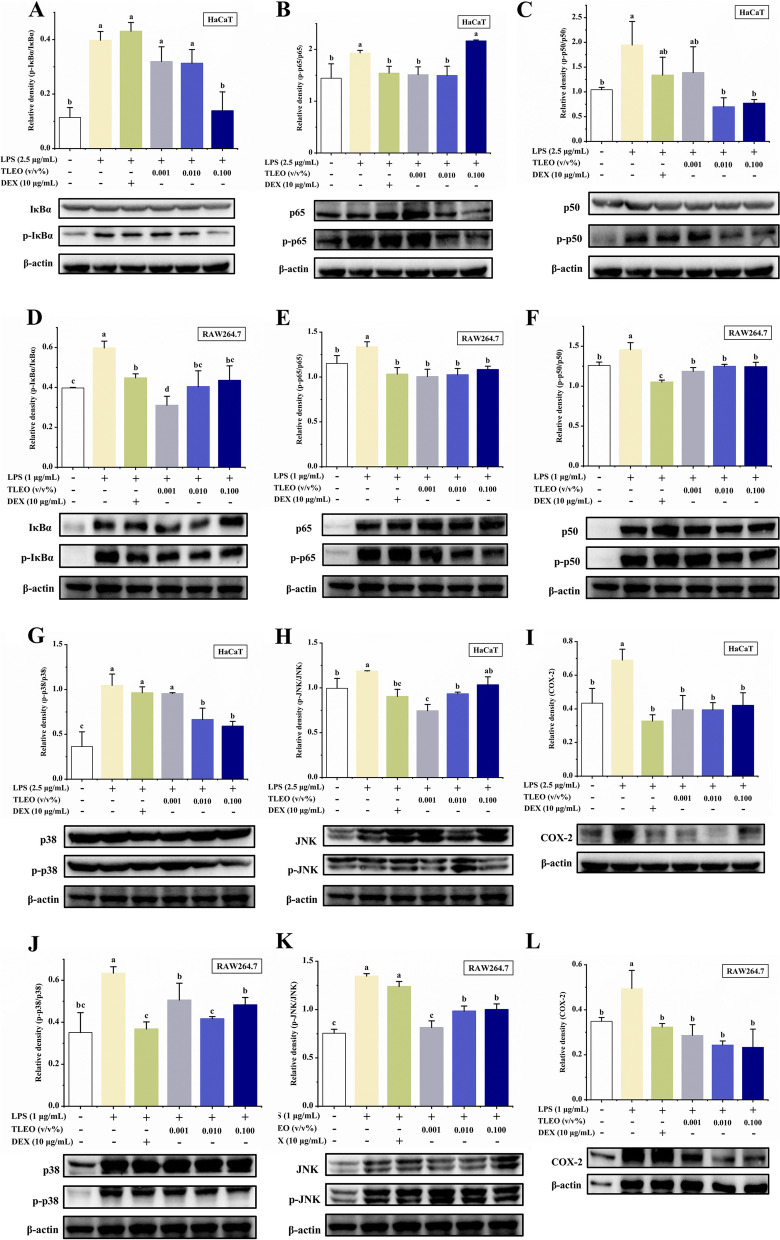
Effects of TLEO on MAPK-NF-κB pathway cascade protein expressions activated in LPS-induced HaCaT and RAW264.7 cells. **A-C:** Effects of TLEO on the phosphorylation of IkB-α, p65, and p50 protein respectively in LPS induced HaCaT cells; **D-F:** Effects of TLEO on the expression of IkB-α, p65, and p50 protein respectively in LPS induced RAW264.7 cells; **G-I:** Effects of TLEO on the phosphorylation of p38MAPK, JNK protein and expression of COX-2 protein in LPS induced HaCaT cells; **J-L:** Effects of TLEO on the phosphorylation of p38MAPK, JNK protein and expression of COX-2 protein in LPS induced RAW264.7 cells. The data are the means ± S.D. (*n* = 3). Statistical analysis was performed by one-way ANOVA with a scheffe’s test. “a”; “b”; “c”; “d” indicates a significant difference (*p* < 0.05) compared with the LPS-treated group

To investigate whether TLEO inhibits NF-κB activation via the MAPK pathway, we used Western blotting to examine the effects of TLEO on the LPS-stimulated phosphorylation of JNK and p38 MAPKs in HaCaT and RAW264.7 cells. As shown in Fig. 4, all TLEO doses significantly suppressed the phosphorylation of the JNK and p38 MAPKs activated by LPS (*p* < 0.05). In the LPS-treated HaCaT model, the inhibition rates of 0.01% and 0.1% TLEO on the phosphorylation expression level of p38 were 36.35% and 43.46%, respectively. The inhibition rate of the phosphorylation expression level of p38 in the DEX treatment group was not significant. While TLEO of 0.001% and 0.01% significantly reduced the phosphorylation level of JNK, by 37.28% and 21.35% (*p* < 0.05), respectively, and the inhibition effect of 0.001% TLEO on JNK phosphorylation level was better than that of DEX (inhibition rate 23.82%). In the inflammatory model of LPS-stimulated RAW264.7, the inhibition rates of 0.001%-0.1% TLEO on p38 phosphorylation levels were 20.15%, 34.23%, and 23.82%, respectively, all slightly lower than those in the DEX treatment group (41.89%). Similarly, TLEO of 0.001%-0.1% showed a significant inhibition effect on JNK phosphorylation levels, with inhibition rates of 39.45%, 26.82%, and 25.61%, respectively (*p* < 0.05). DEX also slightly reduced the expression compared to that of the LPS group, although its effect was not as strong as that of TLEO.

**Fig. 5:**
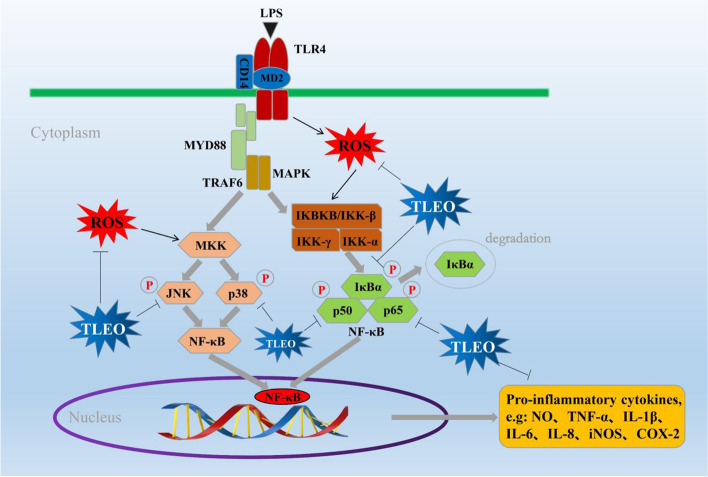
Schematic representation of proposed mechanism that is downregulated by TLEO to reduce inflammation and oxidative stress

Since the phosphorylation of IκB, p50, and p65 and its subsequent degradation are essential steps in NF-κB activation by LPS, we examined the effect of TLEO on these processes by extensive Western blot analysis. Our results showed that 0.1% TLEO decreased the phosphorylation and degradation of IκBα and p50 but promoted p65 phosphorylation in the LPS-activated HaCaT cells (Fig. 4), the inhibition rates of 0.1% TLEO on IκBα and p50 phosphorylation levels reached 65.10% and 60.44% (*p* < 0.05). However, in the LPS-treated RAW264.7 cells, all TLEOs had almost the same inhibitory effects on the phosphorylation of these three NF-κB proteins. The inhibition rates of 0.001%-0.1% TLEO on IκBα, p65, and p50 phosphorylation levels ranged from 25%-50%, 15%-25%, and 10%-20%, respectively. The inhibition rate of the phosphorylation level of IκBα in the 0.001% TLEO treatment group (48.05%) was significantly higher than that in the DEX treatment group (25.05%) (*p* < 0.05). The inhibition rates of the phosphorylation level of p65 in the 0.001%-0.1% TLEO treatment group were 24.80%, 23.30%, and 18.95%, respectively, which was similar to that of the DEX treatment group (22.75%). The inhibition rates of the phosphorylation level of p50 in the 0.001%-0.1% TLEO treatment group were 18.59%, 14.29%, and 14.57%, respectively, significantly lower than the DEX treatment group (27.79%) (*p* < 0.05). However, in both LPS-stimulated cell lines, DEX had slight or no effects on the downregulation of NF-κB pathway proteins (Fig. 5).

### Molecular docking analysis of TLEO components with pathway proteins

The predicted ADME properties for all TLEO components are presented in Supplementary Table S1. Linalyl acetate, linalool, lavandulol acetate, cis-beta-ocimene, 3-octen-3yl acetate, caryophyllene, terpinen-4-ol, (4E, 6E) allocimene, beta-ocimene, geranyl acetate, and b-myrcene were chosen as ligands for molecular docking based on GC‒MS results (> 1%). Mouse IKB-α and JNK proteins were constructed and validated for use (see Supplementary Table S2 and Fig. S2).

**Fig. 6:**
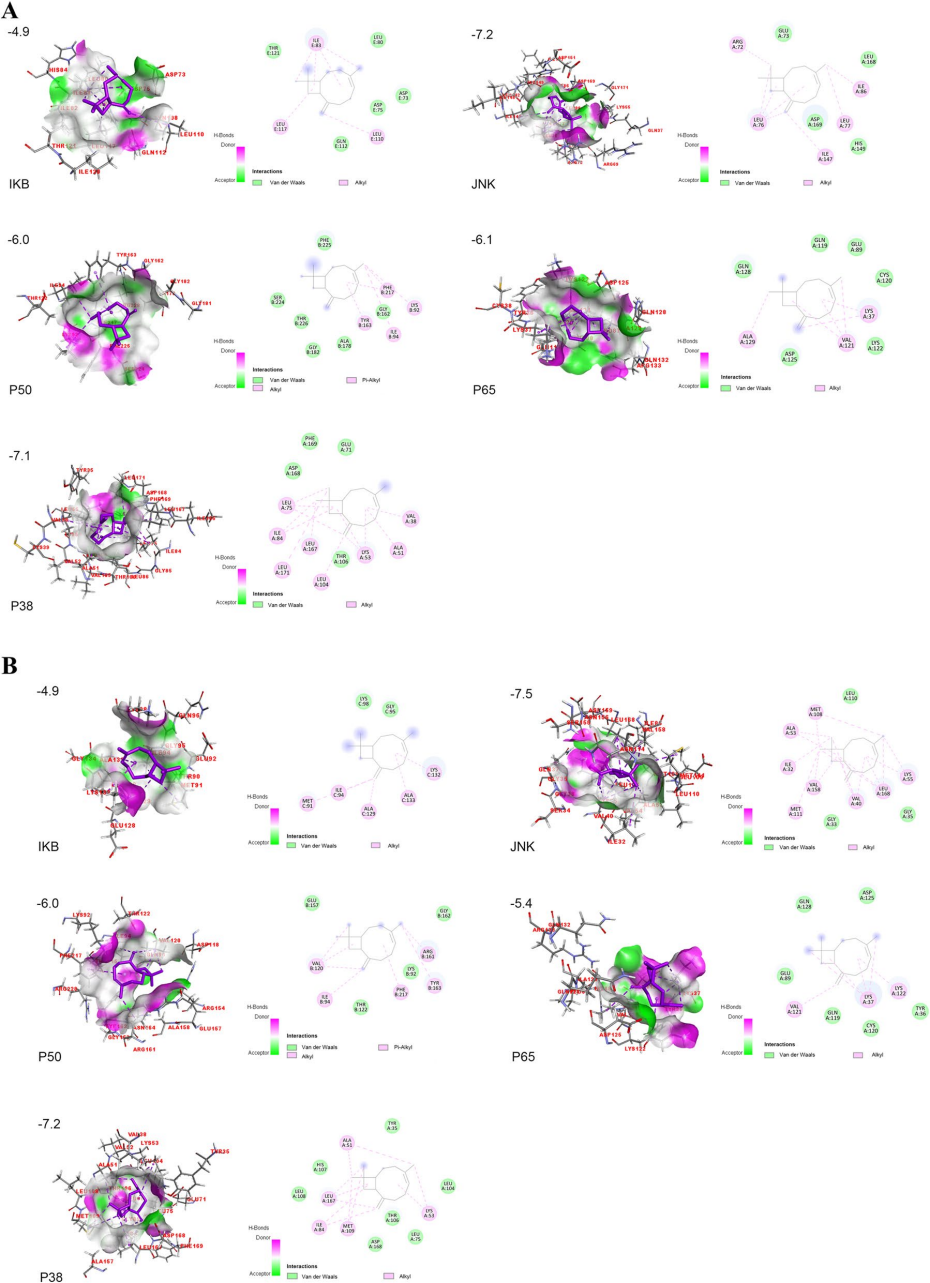
Molecular docking of carphyllene with pathway proteins. **A:** Human proteins IKB-α, JNK, p50, p65, and p38MAPK; **B:** Mice proteins IKB-α, JNK, p50, p65, and p38MAPK

Overall, caryophyllene had the best result for all the proteins in humans and mice followed by geranyl acetate and lavadulol acetate (Fig. 6 and Table 3). This component is bound to human JNK and p38 proteins with affinities of -7.2 and -7.1 kcal/mol, respectively. All the components had weak interactions with IKB-α (> -5.0). The expression of p65 also presented a higher energy requirement (affinity > -5.0) except for caryophyllene. Lavandulol acetate interacted with human p50 at -6.2 kcal/mol. However, for mouse p50, caryophyllene showed the best output of -6.0 kcal/mol binding affinity (see Supplementary Tables S3, S4, S5, S6, S7 and Fig. S3, S4, S5, S6, S7).

**Table 3:** Molecular docking of selective components with pathway proteins

Compound	IKB-α		JNK		p50		p65		p38	
	**Human**	**Mice**	**Human**	**Mice**	**Human**	**Mice**	**Human**	**Mice**	**Human**	**Mice**
Linalyl acetate	-4.1	-4.3	**-6.3**	**-6.3**	-5.8	-5.2	-5.0	-4.7	-5.5	-5.6
linalool	-3.9	-4.2	-5.6	-5.2	**-5.8**	**-5.2**	-5.0	-4.6	-5.1	-5.2
Lavandulol acetate	-4.2	-4.4	**-6.4**	**-6.2**	-6.2	-5.6	-5.2	-5.1	-5.8	-5.7
cis-β-Ocimene	-3.9	-4.1	**-6.2**	-5.7	**-5.8**	-5.0	-5.0	-4.4	-5.2	-4.7
1-Octen-3-yl-acetate	-3.8	-3.9	**-5.6**	**-5.4**	-5.1	-4.6	-4.6	-4.3	-5.0	-5.1
Caryophyllene	-4.9	-4.9	**-7.2**	**-7.5**	-6.0	-6.0	-6.1	-5.4	**-7.1**	**-7.2**
Terpinen-4-ol	-4.2	-4.5	**-6.1**	**-5.5**	-6.0	-5.1	-5.3	-5.0	-5.6	**-5.5**
(4E,6E)-Alloocimene	-4.2	-4.3	**-6.5**	**-6.0**	-6.1	-5.1	-5.5	-4.8	-5.2	-5.3
β-Ocimene	-4.0	-4.0	**-6.4**	**-5.9**	-5.8	-4.5	-4.9	-4.6	-5.1	-4.9
Geranyl acetate	-4.5	-4.4	**-6.7**	**-6.4**	-6.1	-5.2	-5.6	-4.6	-5.8	-5.7
β-Myrcene	-4.0	-4.2	**-6.1**	**-5.6**	-5.6	-4.9	-4.9	-4.2	-5.0	-4.8

## Discussion

The major components in TLEO are linalool and linalyl acetate (29.48% and 40.97%, respectively), similar to other *L. angustifolia* species. However, some components that are commonly found in trace amounts (< 0.9%) in other LEOs were found to be significantly higher in TLEO, such as lavandulol acetate (4.77%), cis-β-ocimene (3.04%), 1-octen-3-yl acetate (2.62%), caryophyllene (2.49%), terpinen-4-ol (2.44%), (4E, 6E)-alloocimene (1.23%), β-ocimene (1.2%), geranyl acetate (1.11%), and β-myrcene (1.1%) [21, 22]. Space radiation is one of the methods used to create superior crop varieties. After exposure to cosmic radiation, the lavender seeds gain genotypic and phenotypic changes by the stress of space radiation and microgravity. Through several generations of culture and selection, germplasms with useful and stable modified characteristics have been obtained [23]. “TaiKhong Blue” lavender is one of the mutated cultivars that exhibit high EO productivity, a long-blooming period, and some content changes in chemical composition [24]. The main components of many essential oils known to have an anti-inflammatory effect are linalool and linalyl acetate [25].

Dexamethasone (DEX), a standard anti-inflammatory drug, was used as a positive control in this study. DEX not only reduces the production of inflammatory cytokines, such as IL-6, TNF-α, and NO, by LPS-induced inflammation [25–27] but also reduces MDA levels in rabbits suffering from oral ulcers [25, 26, 28]. However, TLEO showed better effects than DEX in average for our analyses.

We induced inflammation in our experiment with lipopolysaccharide (LPS), an endotoxin produced by *E.coli*. Through the toll-like receptor 4 (TLR4), LPS enters the body and activates macrophages, neutrophils, and endothelial cells like keratinocytes, which results in the production of numerous inflammatory factors, and oxidative stress markers and leads to organ dysfunction (Caroline, 2015). So, the downregulation of the TLR4 pathway is one of the strategies to reduce inflammation in cells. We studied the most prominent pathway of inflammation, which is MAPK-NF-κB signaling. In our present study, TLEO showed good inhibition of the excessive production of NO and ROS in the LPS-treated HaCaT cells and RAW264.7 murine macrophages. This ROS-alleviating effect was also observed by other researchers. Kozics et al. (2017) found that pretreatment with EO from *L. angustifolia* could inhibit the oxidant stress induced by H_2_O_2_ in human hepatoma cells (HepG2) [25, 27, 29]. EO from *L. stoechas* could also significantly decrease the production of MDA and increase the activities of SOD and CAT to inhibit alloxan-induced oxidative stress in rats [30].

The ROS alleviating effect of TLEO may be due to the downregulation of key proteins in MAPK-NF-κB signaling (Fig. 5). In the present study, TLEO lowered the expression of the p38MAPK, JNK, IkB-α, p65, and p50 proteins and subsequently caused a significant reduction in TNF-α, IL-1β and IL-6 cytokine expression at both the mRNA and protein levels. Few studies refer to the inhibition of the NF-κB pathway-related and cytokine expression by LEOs, which is similar to our results. For example, in a myocardial infarction rat model, *L. angustifolia* EO significantly reduced TNF-α and COX-2 expression *by* inhibiting NF-кB activity [31]. Baker et al. (2012) applied *L. x intermedia* essential oil against *C. rodentium*-induced colitis in C57BL/6 mice, and the expression of typical inflammatory factors, such as TNF-α, IFN-γ, IL-22, and iNOS, was remarkably downregulated [32].

Our preliminary experiments showed that TLEO exhibited a better anti-inflammatory effect than essential oils from ‘French blue’ lavender and 701 lavender (types available in Xinjiang), two commonly used varieties in Xinjiang’s industry (Fig. S8). The compositional variation might be the key to explaining differences in their activity, but the compounds that are responsible for the difference remain unknown. Molecular docking might shed light on this issue.

Considering all 11 ligands, IKB-α protein from both humans and mice exerted the weakest effect, and JNK showed the highest effect. Caryophyllene, lavandulol acetate, and geranyl acetate were the top 3 ligands that bound to proteins in the MAPK-NF-κB signaling pathway with the lowest affinities. These 3 compounds are relatively abundant in TLEO and are present in trace amounts in other common LEOs. In addition to these compounds, linalyl acetate, as a major component in almost all lavender essential oils, also maintained a relatively high binding affinity (< -5.0 kcal/mol), while 1-octen-3-yl-acetate had the weakest interaction.

Caryophyllene interacted with JNK and p38 in both humans and mice with the lowest affinities > -7.0 kcal/mol. The human JNK protein interacted with caryophyllene by five alkyl bonds at Arg 72, Leu 76–77, Ile 86, and Ile 147 and four van der Waals bonds. The ligand shared eight alkyl bonds as well as four van der Waals bonds with human p38 amino acids ranging from 38 to 171 and was enriched with leucine residues. These properties make p38-caryophyllene more stable than JNK-caryophyllene in humans. However, in mice, JNK and p38 showed the same number of alkyl bonds, varying in the quantity of van der Waals bonds. As p50 and p65 work as dimers to activate the pathway, interacting with one of them could cause changes in the signal. Caryophyllene interacted with both human and mouse p50 with an affinity of -6.0 kcal/mol. These molecules shared alkyl and van der Waals bonds with five common residues: Lys 92, Ile 94, Gly 162, Tyr 163, and Phe 217 (Table 4). The next three best ligand candidates were lavandulol acetate, geranyl acetate, and linalyl acetate (Table 3). All three interacted best with JNK protein from both organisms sharing more than 8 alkyl bonds. Lavandulol acetate shared two conventional hydrogen bonds (Asn 114, Thr 183) with human JNK protein, while geranyl acetate (Met 111) and linalyl acetate (Thr 183) each shared one. For mouse JNK, the interacting bonds for geranyl acetate were conventional hydrogen bonds at Met 111, carbon-hydrogen bonds at Leu 110, alkyl bonds at Ile 86, Leu 106, Met 108, and Leu 168, and five van der Waals bonds at Ile 32, Ala 53, Leu 88, Glu 109, and Val 158 (see Supplementary data). However, there is also experimental evidence of the anti-inflammatory effects of these compounds. A study on a rat skin wound excision model indicated that caryophyllene reduced IFN-γ, IL-1β, IL-6, and TNF-α levels and participated in wound healing by an anti-inflammatory mechanism [33]. Geranyl acetate can decrease inflammation and relieve pain in different experimental models. Linalyl acetate also exhibited anti-inflammatory effects on carrageenan-induced edema in rats [34]. Linalool inhibits the generation and expression of inflammatory mediators (TNF-α, IL-6, IL-1, NO, and PGE2) by blocking the NF-kB and MAPK signaling pathways and activating the Nrf2/HO-1 signaling pathway [35–40].

**Table 4:** Interacting residues of pathway proteins with Caryophyllene

Organism	Proteins	Binding Affinity (kcal/mol)	Interaction	
			**H/Alkyl/Covalent bonds**	**Van der wales bond**
**Human**	IKB-α	-4.9	Ile 83, Leu 110, Leu 117	Asp 73, Asp 75, Leu 80, Gln 112, Thr 121
	JNK	**-7.2**	Arg 72, Leu 76, Leu 77, Ile 86, Ile 147	Glu 73, His 149, Leu 168, Asp 169
	p50	-6.0	Lys 92, Ile 94, Tyr 163, Phe 217	Gly 162, Ala 178, Gly 182, Ser 224, Phe 225, Thr 226
	p65	-6.1	Lys 37, Val 121, Ala 129	Glu 89, Gln 119, Cys 120, Lys 122, Asp 125, Gln 128
	p38	**-7.1**	Val 38, Ala 51, LyS 53, Leu 75, Ile 84, Leu 104, Leu 167, Leu 171	Glu 71, Thr 106, Asp 168, Phe 169
**Mice**	IKB-α	-4.9	Met 91, Ile 94, Ala 129, Lys 132, Ala 133	Gly 95, Lys 98
	JNK	**-7.5**	Ile 32, Val 40, Ala 53, Lys 55, Met 108, Met 111, Val 158, Leu 168	Gly 33, Gly 35, Leu 110
	p50	-6.0	Ile 94, Val 120, Tyr 163, Arg 161, Phe 217	Lys 92, Thr 122, Glu 157, Gly 162
	p65	-5.4	Lys 37, Val 121, Lys 122	Tyr 36, Glu 89, Gln 119, Cys 120, Asp 125, Gln 128
	p38	**-7.2**	Lys 53, Ala 51, Ile 84, Met 109, Leu 167	Tyr 35, Leu 75, Leu 104, Thr 106, His 107, Leu 108, Asp 168

Therefore, our overall molecular docking results suggested that the relatively high amounts of caryophyllene, lavandulol acetate, and geranyl acetate in TLEO might be the key to explaining the better anti-inflammatory effect of TLEO than other lavender essential oils and that linalyl acetate is an important contributor to the anti-inflammatory activity of all lavender essential oils. We further predicted the ADME properties of TLEO components, and the results showed that all the components were safe for use as food and medication.

## Conclusions

In summary, molecular biology and molecular docking analysis provide us with a combined perspective on the anti-inflammatory potential of TLEO. Our overall findings proved to have significance for the further development and utilization of TLEO in food, medicine, and cosmetics. The use of TLEO in trace amounts in everyday life could also have preventive potential, which needs more investigation. As other LEOs are already in use for cosmetics, ointment products, and food flavors, TLEO could be introduced as such, and its clinical implementation can be planned in the future to determine its effects on humans. Furthermore, the utilization of TLEO components individually or in other composition ratios may be a possible future area of study.     

## Supplementary Information

**Additional file 1:** **Fig. S1.** 11 Ligands with their structure. **Figure S2.** Ramachandran plot for Mice IkB-α(A) and JNK(B) protein from swiss model. **Fig. S3.** Molecular docking of TLEO components with Human and Mice IkB-αprotein. **Fig. S4.** Moleculardocking of TLEO components with Human and Mice JNK protein.**Fig. S5.** Molecular docking of TLEO components withHuman and Mice p50 protein. **Fig. S6.** Molecular docking of TLEO components with Human and Mice p65 protein. **Fig. S7.** Moleculardocking of TLEO components with Human and Mice p38 protein.**Fig. S8.** Effects of TLEO on mRNA andprotein level of pro-inflammatory cytokines IL-6 by LPS-induced RAW264.7cells. The data are the means ± S.D (*n* = 3). Statistical analysis was performedby one-way ANOVA with a scheffe’s test. “*” and “#”  indicate significant difference (*p <0.05*) compared with the LPS-treated group. **Fig. S9.** Total ionchromatography from GC-MS analysis of TLEO. **Table S1.** ADME analysis of TLEOcomponents. **Table S2.** Validation of mice protein IKB-α and JNK. **Table S3.** Molecular docking of major TLEO componentswith protein IKB-α. **Table S4.** Molecular docking of major TLEO componentswith protein JNK. **Table S5.** Molecular docking of major TLEO components withprotein p50. **Table S6.** Molecular docking of major TLEO components with protein p65. T**able S7.**  Molecular docking of major TLEO components with protein p38.

## Data Availability

The data used to support the findings of this study are available from the corresponding author upon request.
