# Supplementary material for: In vitro and in silico analysis of ‘Taikong blue’ lavender essential oil in LPS-induced HaCaT cells and RAW264.7 murine macrophages
Source: BMC Complement Med Ther. 2022 Dec 6;22:324. doi: 10.1186/s12906-022-03800-0 (PMC9727978; doi:10.1186/s12906-022-03800-0)
Supplement: Supplementary file 1 — Additional file 1: Fig. S1. 11 Ligands with their structure. Figure S2. Ramachandran plot for Mice IkB-α(A) and JNK(B) protein from swiss model. Fig. S3. Molecular docking of TLEO components with Human and Mice IkB-αprotein. Fig. S4. Moleculardocking of TLEO components with Human and Mice JNK protein.Fig. S5. Molecular docking of TLEO components withHuman and Mice p50 protein. Fig. S6. Molecular docking of TLEO components with Human and Mice p65 protein. Fig. S7. Moleculardocking of TLEO components with Human and Mice p38 protein.Fig. S8. Effects of TLEO on mRNA andprotein level of pro-inflammatory cytokines IL-6 by LPS-induced RAW264.7cells. The data are the means ± S.D (n = 3). Statistical analysis was performedby one-way ANOVA with a scheffe’s test. “*” and “#” indicate significant difference (p <0.05) compared with the LPS-treated group. Fig. S9. Total ionchromatography from GC-MS analysis of TLEO. Table S1. ADME analysis of TLEOcomponents. Table S2. Validation of mice protein IKB-α and JNK. Table S3. Molecular docking of major TLEO componentswith protein IKB-α. Table S4. Molecular docking of major TLEO componentswith protein JNK. Table S5. Molecular docking of major TLEO components withprotein p50. Table S6. Molecular docking of major TLEO components with protein p65. Table S7. Molecular docking of major TLEO components with protein p38. [file 12906_2022_3800_MOESM1_ESM.docx]

**Table S1: ADME analysis of TLEO components**

| **Compound** | **Formula** | **MW** | **TPSA** | **iLOGP** | **Consensus Log P** | **ESOL Solubility (mg/ml)** | **ESOL Class** | **GI absorption** | **BBB permeant** | **log Kp (cm/s)** | **Lipinski #violations** |
| --- | --- | --- | --- | --- | --- | --- | --- | --- | --- | --- | --- |
| α-Pinene | C_10_H_16_ | 136.23 | 0 | 2.63 | 3.44 | 4.24E-02 | Soluble | Low | Yes | -3.95 | 1 |
| Camphene | C_10_H_16_ | 136.23 | 0 | 2.58 | 3.43 | 6.18E-02 | Soluble | Low | Yes | -4.13 | 1 |
| Butyl acetate | C_6_H_12_O_2_ | 116.16 | 26.3 | 2.17 | 1.53 | 4.44E+00 | Very soluble | High | Yes | -5.74 | 0 |
| β-Pinene | C_10_H_16_ | 136.23 | 0 | 2.59 | 3.42 | 6.74E-02 | Soluble | Low | Yes | -4.18 | 1 |
| Sabinen | C_10_H_16_ | 136.23 | 0 | 2.65 | 3.25 | 3.71E-01 | Soluble | Low | Yes | -4.94 | 1 |
| 3-Carene | C_10_H_16_ | 136.23 | 0 | 2.63 | 3.42 | 4.90E-02 | Soluble | Low | Yes | -4.02 | 1 |
| β-Myrcene | C_10_H_16_ | 136.23 | 0 | 2.89 | 3.43 | 1.22E-01 | Soluble | Low | Yes | -4.17 | 0 |
| α-Terpine | C_10_H_16_ | 136.23 | 0 | 2.7 | 3.3 | 6.89E-02 | Soluble | Low | Yes | -4.11 | 0 |
| D-Limonene | C_10_H_16_ | 136.23 | 0 | 2.72 | 3.37 | 4.33E-02 | Soluble | Low | Yes | -3.89 | 0 |
| Eucalyptol | C_10_H_18_O | 154.25 | 9.23 | 2.58 | 2.67 | 4.63E-01 | Soluble | High | Yes | -5.3 | 0 |
| Butyl butylate | C_8_H_16_O_2_ | 144.21 | 26.3 | 2.68 | 2.17 | 2.80E+00 | Very soluble | High | Yes | -5.63 | 0 |
| cis-β-Ocimene | C_10_H_16_ | 136.23 | 0 | 2.8 | 3.4 | 9.20E-02 | Soluble | Low | Yes | -4.11 | 0 |
| β-Ocimene | C_10_H_16_ | 136.23 | 0 | 2.8 | 3.4 | 9.20E-02 | Soluble | Low | Yes | -4.11 | 0 |
| 3-Octanone | C_8_H_16_O | 128.21 | 17.07 | 2.39 | 2.33 | 2.16E+00 | Very soluble | High | Yes | -5.43 | 0 |
| Hexyl acetate | C_8_H_16_O_2_ | 144.21 | 26.3 | 2.66 | 2.22 | 1.98E+00 | Very soluble | High | Yes | -5.46 | 0 |
| Terpinolene | C_10_H_16_ | 136.23 | 0 | 2.71 | 3.4 | 4.30E-02 | Soluble | Low | Yes | -3.96 | 0 |
| Hexyl isobutyrate | C_10_H_20_O_2_ | 172.26 | 26.3 | 3.12 | 2.91 | 4.08E-01 | Soluble | High | Yes | -4.89 | 0 |
| Hexyl formate | C_7_H_14_O_2_ | 130.18 | 26.3 | 0 | 1.5 | 2.06E+00 | Very soluble | High | Yes | -5.35 | 0 |
| (4E,6E)-Alloocimene | C_10_H_16_ | 136.23 | 0 | 2.93 | 3.37 | 8.62E-02 | Soluble | Low | Yes | -4.15 | 0 |
| 1-Octen-3-yl, acetate | C_10_H_18_O_2_ | 170.25 | 26.3 | 2.58 | 2.69 | 6.69E-01 | Soluble | High | Yes | -5.12 | 0 |
| Hexyl butanoate | C_10_H_20_O_2_ | 172.26 | 26.3 | 3.01 | 2.91 | 6.44E-01 | Soluble | High | Yes | -5.04 | 0 |
| Linalool oxide | C_10_H_18_O_2_ | 170.25 | 32.76 | 2.56 | 1.99 | 2.92E+00 | Very soluble | High | Yes | -6.06 | 0 |
| 1-Octen-3-ol | C_22_H_40_O_7_ | 416.55 | 132.13 | 3.1 | 4.43 | 2.64E-03 | Moderately soluble | Low | No | -4.23 | 0 |
| Linalool oxide | C_10_H_18_O_2_ | 170.25 | 32.76 | 2.56 | 1.99 | 2.92E+00 | Very soluble | High | Yes | -6.06 | 0 |
| α-Copaene | C_15_H_24_ | 204.35 | 0 | 3.4 | 4.3 | 2.84E-02 | Soluble | Low | Yes | -4.37 | 1 |
| Camphor | C_10_H_16_O | 152.23 | 17.07 | 2.12 | 2.37 | 1.04E+00 | Soluble | High | Yes | -5.67 | 0 |
| Linalool | C_10_H_18_O | 154.25 | 20.23 | 2.7 | 2.66 | 6.09E-01 | Soluble | High | Yes | -5.13 | 0 |
| Linalyl acetate | C_12_H_20_O_2_ | 196.29 | 26.3 | 3.08 | 3.24 | 1.43E-01 | Soluble | High | Yes | -4.71 | 0 |
| α-Santalene | C_15_H_24_ | 204.35 | 0 | 3.43 | 4.59 | 1.32E-02 | Moderately soluble | Low | No | -3.85 | 1 |
| Bornyl acetate | C_12_H_20_O_2_ | 196.29 | 26.3 | 2.5 | 3 | 4.56E-02 | Soluble | High | Yes | -4.44 | 0 |
| β-Copaene | C_15_H_24_ | 204.35 | 0 | 3.35 | 4.4 | 2.00E-02 | Moderately soluble | Low | Yes | -4.2 | 1 |
| Caryophyllene | C_15_H_24_ | 204.35 | 0 | 3.29 | 4.24 | 2.78E-02 | Soluble | Low | No | -4.44 | 1 |
| Terpinen-4-ol | C_10_H_18_O | 154.25 | 20.23 | 2.51 | 2.6 | 2.54E-01 | Soluble | High | Yes | -4.93 | 0 |
| Lavandulol acetate | C_12_H_20_O_3_ | 212.29 | 46.53 | 2.48 | 2.54 | 4.23E-01 | Soluble | High | Yes | -5.41 | 0 |
| cis-β-Farnesene | C_15_H_24_ | 204.35 | 0 | 3.86 | 4.97 | 7.35E-03 | Moderately soluble | Low | No | -3.27 | 1 |
| trans-β-Farnesene | C_15_H_24_ | 204.35 | 0 | 3.86 | 4.97 | 7.35E-03 | Moderately soluble | Low | No | -3.27 | 1 |
| Humulene | C_15_H_24_ | 204.35 | 0 | 3.27 | 4.26 | 2.17E-02 | Soluble | Low | No | -4.32 | 1 |
| Lavandulol | C_10_H_18_O | 154.25 | 20.23 | 2.51 | 2.6 | 5.67E-01 | Soluble | High | Yes | -5.1 | 0 |
| Cryptone | C_9_H_14_O | 138.21 | 17.07 | 2.05 | 2.06 | 1.73E+00 | Very soluble | High | Yes | -5.71 | 0 |
| α-Terpineol | C_10_H_18_O | 154.25 | 20.23 | 2.51 | 2.58 | 2.10E-01 | Soluble | High | Yes | -4.83 | 0 |
| Borneol | C_10_H_18_O | 154.25 | 20.23 | 2.29 | 2.38 | 4.77E-01 | Soluble | High | Yes | -5.31 | 0 |
| D-Germacrene | C_15_H_24_ | 204.35 | 0 | 3.32 | 4.3 | 1.92E-02 | Moderately soluble | Low | No | -4.18 | 1 |
| Nerol acetate | C_12_H_20_O_2_ | 196.29 | 26.3 | 2.83 | 3.21 | 1.22E-01 | Soluble | High | Yes | -4.63 | 0 |
| Carvone | C_10_H_14_O | 150.22 | 17.07 | 2.27 | 2.44 | 5.81E-01 | Soluble | High | Yes | -5.29 | 0 |
| Geranyl acetate | C_12_H_20_O_2_ | 196.29 | 26.3 | 2.83 | 3.21 | 1.22E-01 | Soluble | High | Yes | -4.63 | 0 |
| Cuminal | C_10_H_12_O | 148.2 | 17.07 | 2.03 | 2.48 | 4.44E-01 | Soluble | High | Yes | -5.52 | 0 |
| Nerol | C_10_H_18_O | 154.25 | 20.23 | 2.75 | 2.78 | 2.59E-01 | Soluble | High | Yes | -4.71 | 0 |
| Guaniol | C_10_H_18_O | 154.25 | 20.23 | 2.75 | 2.78 | 2.59E-01 | Soluble | High | Yes | -4.71 | 0 |
| p-Cymen-8-ol | C_10_H_14_O | 150.22 | 20.23 | 2.17 | 2.25 | 6.52E-01 | Soluble | High | Yes | -5.8 | 0 |
| Caryophyllene oxide | C_15_H_24_O | 220.35 | 12.53 | 3.15 | 3.68 | 7.84E-02 | Soluble | High | Yes | -5.12 | 0 |
| Cuminol | C_10_H_14_O | 150.22 | 20.23 | 2.33 | 2.39 | 4.57E-01 | Soluble | High | Yes | -5.55 | 0 |
| T-Cadinol | C_15_H_26_O | 222.37 | 20.23 | 3.15 | 3.43 | 1.23E-01 | Soluble | High | Yes | -5.29 | 0 |
| Coumarin | C_9_H_6_O_2_ | 146.14 | 30.21 | 1.75 | 1.82 | 7.42E-01 | Soluble | High | Yes | -6.2 | 0 |


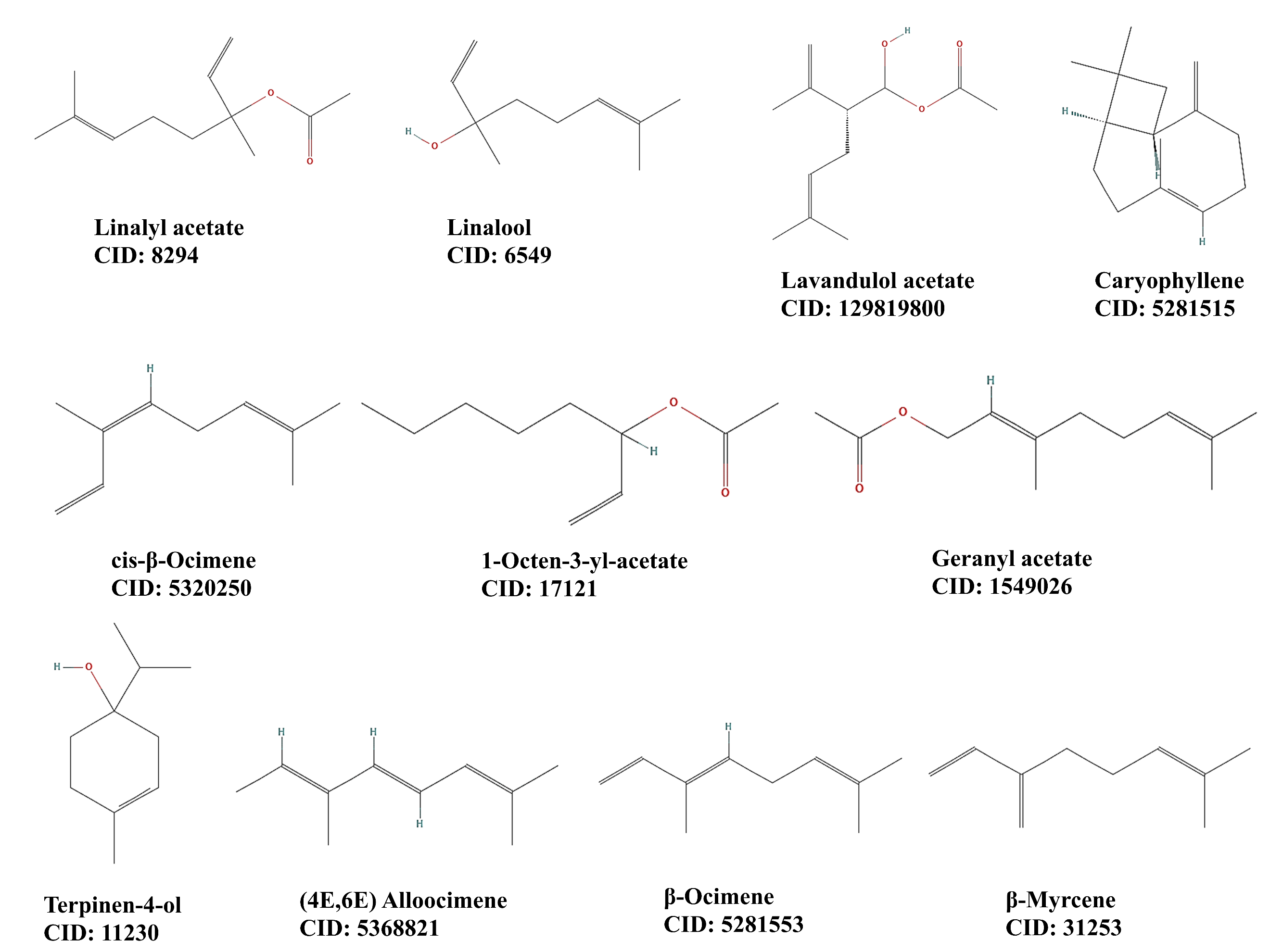


**Fig. S1: 11 Ligands with their structure**

**Table S2: Validation of mice protein IKB-α and JNK**

| **Mice**  **Protein** | **Model** | **ERRAT** | **Verify 3D(%)** | **Favored region(%)** | **Allowed Region(%)** | **Non-allowed region(%)** |
| --- | --- | --- | --- | --- | --- | --- |
| IkB-α | Alphafold | 98.3871 | 64.01 | 81.0 | 19.1 | 0 |
|  | Swiss-Model | 96.0396 | 86.73 | 89.1 | 10.9 | 0 |
| JNK | Alphafold | 97.9943 | 70.25 | 86.9 | 11.3 | 1.8 |
|  | Swiss-Model | 91.4373 | 81.46 | 91.0 | 8.4 | 0.6 |


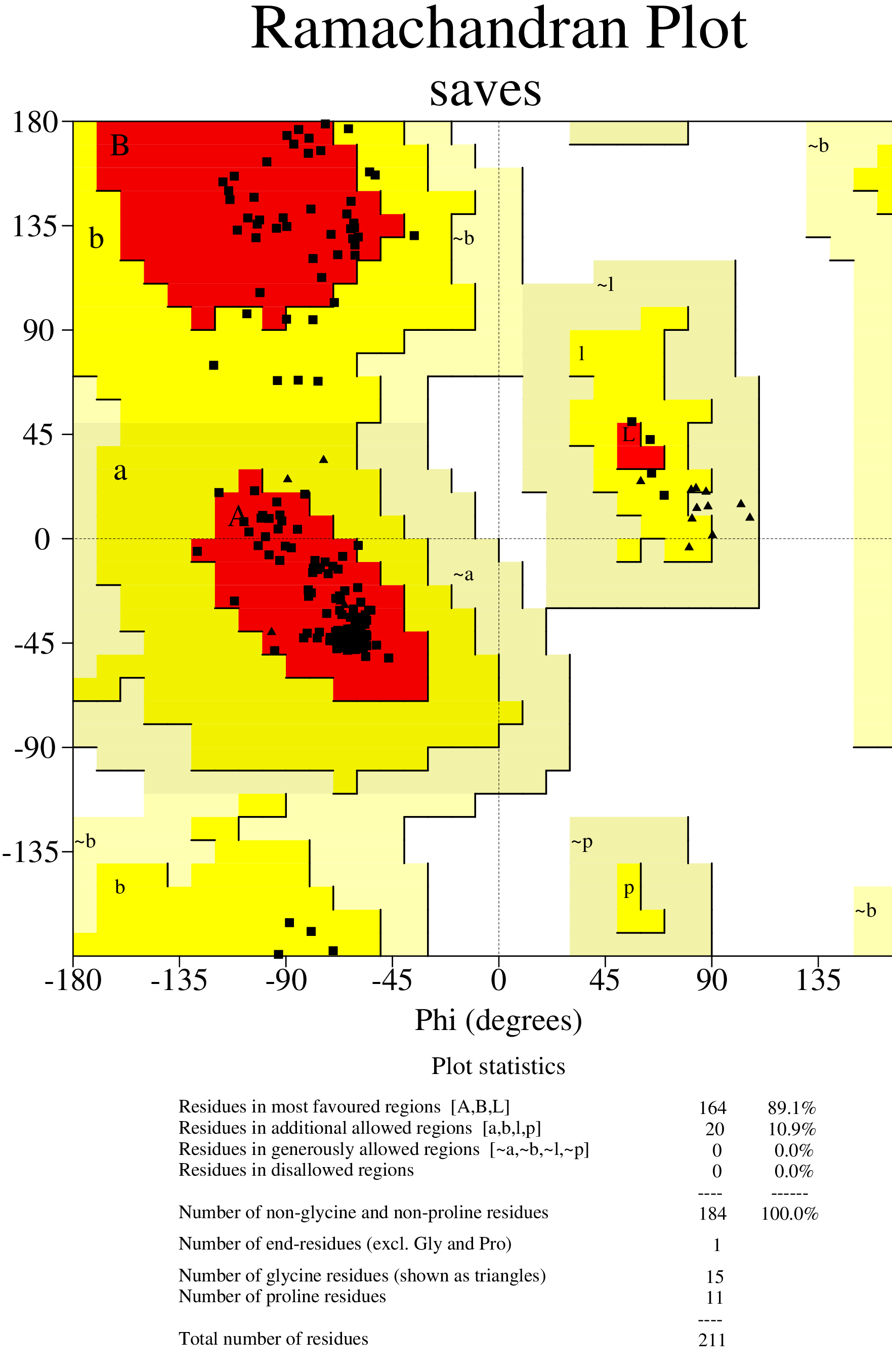


**A**


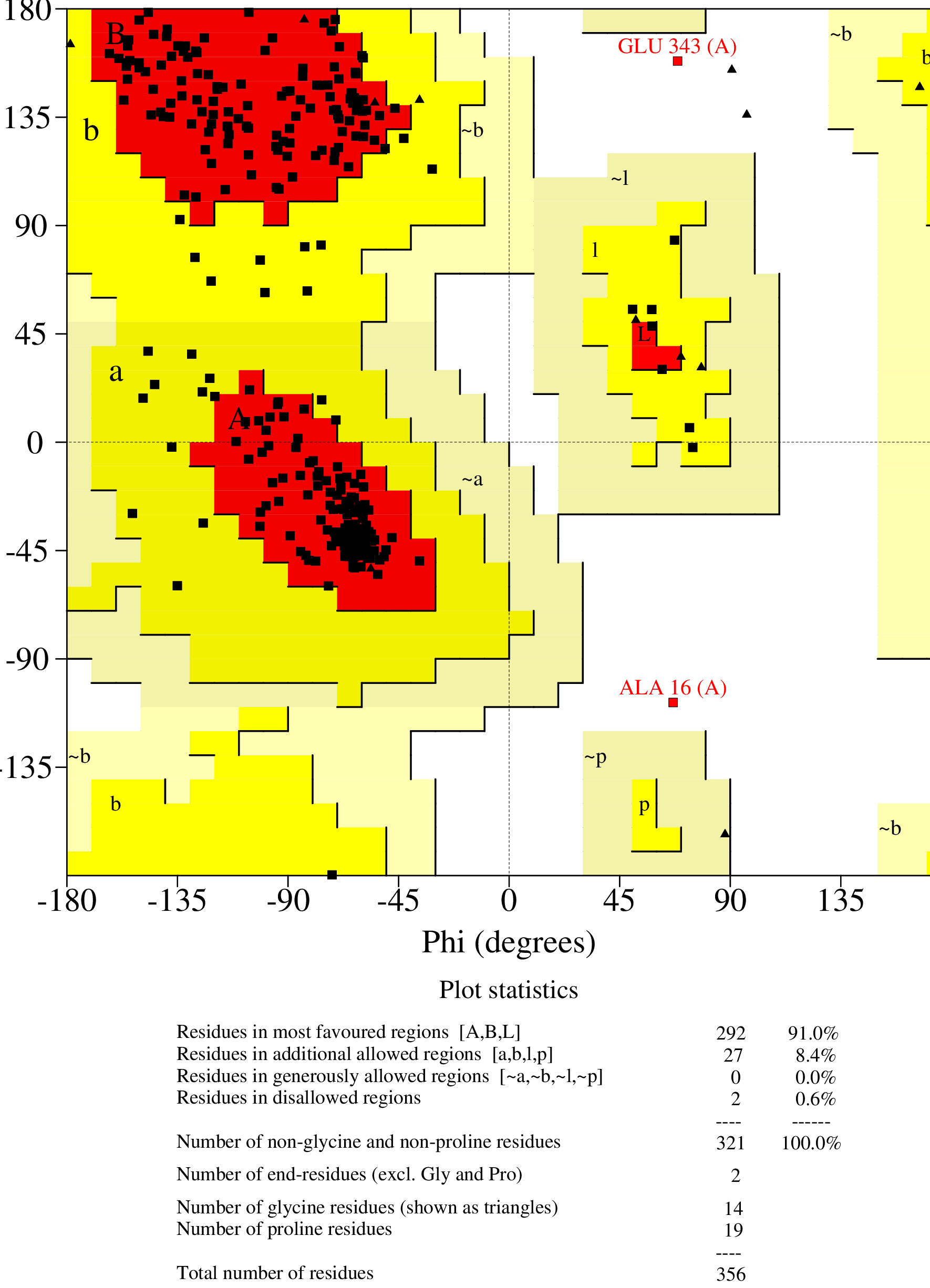


**B**

**Figure S2:** **Ramachandran plot for Mice IkB-α(A) and JNK(B) protein from swiss model**

**Table S3: Molecular docking of major TLEO components with protein IKB-α**

| **Organism** | **Compound** | **Binding Affinity**  **(kcal/mol)** | **Interaction**  **H/Alkyl/Covalent bonds** | **Van der wales bond** |
| --- | --- | --- | --- | --- |
| Human | Linalyl acetate | -4.1 | Leu176, Lys 177, Ala 211, | Thr 179, Thr 185, Gly 206, Ala 207, Asp 208, Gln 212 |
|  | linalool | -3.9 | Lys 177, Ala 211, Gln 212 | Leu 176, Thr 185, Asp 208, Asn 210, Arg 245 |
|  | Lavandulol acetate | -4.2 | Arg 140, Lys 177, Ala 178, Thr 179 | Ser 174 |
|  | cis-β-Ocimene | -3.9 | His 173, Leu 176, Lys 177, Ala 211 | Thr 179, Thr 185, Gly 206, Ala 207, Asp 208, Gln 212 |
|  | 1-Octen-3-yl-acetate | -3.8 | Lys 177, Pro 214 | Leu 176, Thr 179, Thr 185, Ala 207, Asp 208, Ala 211, Gln 212 |
|  | Terpinen-4-ol | -4.2 | Asp 75, Ile 83, Leu 117, Ile 120 | Asp 73, Asn 108, Leu 110, Gln 112 |
|  | (4E,6E) Alloocimene | -4.2 | His 173, Leu 176, Lys 177, Ala 211 | Thr 179, Thr 185, Gly 206, Ala 207, Asp 208, Gln 212 |
|  | β-Ocimene | -4.0 | His 173, Leu 176, Lys 177, Ala 211 | Thr 185, Gly 206, Ala 207, Asp 208 |
|  | Geranyl acetate | -4.5 | Asn 180, His 184, Ile 192, His 193, Leu 223, Leu 227 | Arg 143, Asn 145, Glu 153, Asn 182, Leu 189, Asp 226 |
|  | β-Myrcene | -4.0 | Ile 83, Leu 110, Leu 117, Ile 120 | Asp 73, Asp 75, Asn 108, Gln 112, Thr 121 |
| Mice | Linalyl acetate | -4.3 | Met 91, Ile 94, Lys 98, Gly 99, Ala 129, Lys 132, Ala 133 | Gly 95 |
|  | linalool | -4.2 | Met 91, Ile 94, Lys 98, Ala 129, Lys 132, Ala 133 | Gly 95, Gly 99 |
|  | Lavandulol acetate | -4.4 | Asp 75, Leu 80, Ile 83, His 84, Leu 117 | Asp 73, His 79, Asn 108, Asn 109, Leu 110, Gln 112 |
|  | cis-β-Ocimene | -4.1 | Met 91, Ile 94, Lys 98, Ala 129, Lys 132, Ala 133 | Gly 95， Gly 99 |
|  | 1-Octen-3-yl-acetate | -3.9 | Met 91, Lys 98, Gly 99, Ala 129, Lys 132, Ala 133 | Ile 94, Gly 95 |
|  | Terpinen-4-ol | -4.5 | Met 91, Ile 94, Ala 129, Lys 132, Ala 133 | Gly 95, Lys 98, Gly 99 |
|  | (4E,6E)-Alloocimene | -4.3 | Met 91, Ile 94, Lys 98, Ala 129, Lys 132, Ala 133 | Gly 95, Gly 99 |
|  | β-Ocimene | -4.0 | Met 91, Ile 94, Lys 98, Ala 129, Lys 132, Ala 133 | Gly 95 |
|  | Geranyl acetate | -4.4 | Ile 83, Leu 110, Leu 117, Ile 120, | Asp 73, Asp 75, Leu 80, His 84, Asn 108, Gln 112, Thr 121 |
|  | β-Myrcene | -4.2 | Met 91, Ile 94, Lys 98, Ala 129, Lys 132, Ala 133 | Gly 95, Gly 99 |


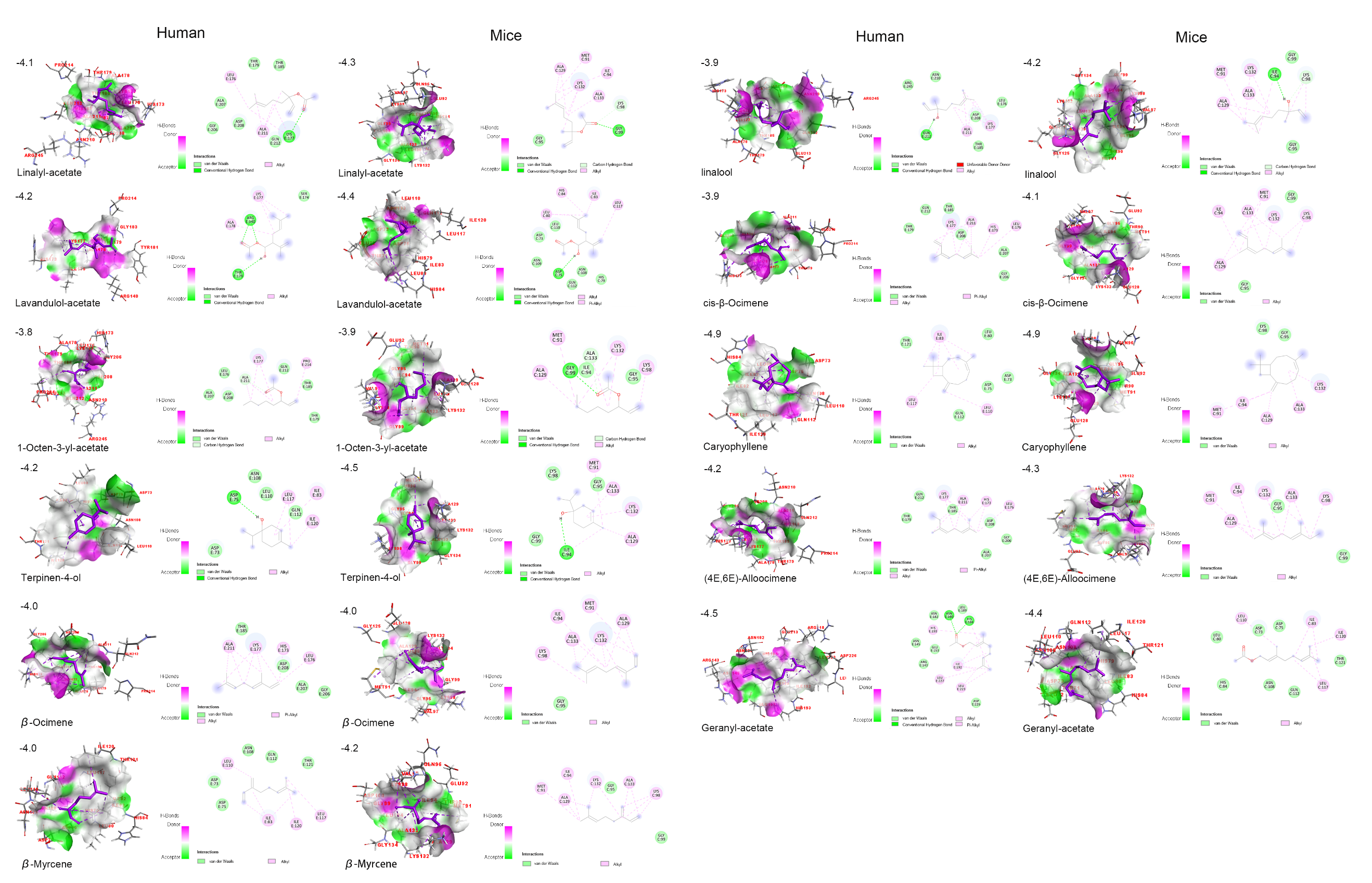


**Fig. S3: Molecular docking of TLEO components with Human and Mice IkB-α protein**

**Table S4: Molecular docking of major TLEO components with protein JNK**

| **Organism** | **Compound** | **Binding Affinity**  **(kcal/mol)** | **Interaction**  **H/Alkyl/Covalent bonds** | **Van der wales bond** |
| --- | --- | --- | --- | --- |
| Human | Linalyl acetate | -6.3 | Ile 32, Val 40, Ala 53, Lys 55, Met 108, Leu 110, Met 111, Val 158, Leu 168, Phe 170, Thr 183 | Asn 114, Ser 155, Asp 169 |
|  | linalool | -5.6 | Ile 32, Val 40, Ala 53, Met 108, Met 111, Val 158, Leu 168, Phe 170 | Glu 109, Leu 110, Thr 183 |
|  | Lavandulol acetate | -6.4 | Val 40, Ala 53, Met 108, Leu 110, Asn 114, Leu 168, Phe 170, Thr 183 | Ile 32, Glu 109, Met 111, Val 158 |
|  | cis-β-Ocimene | -6.2 | Val 40, Lys 55, Ile 86, Leu 88, Leu 106, Met 108, Leu 168, Phe 170 | Glu 73, Val 107, Asp 169 |
|  | 1-Octen-3-yl-acetate | -5.6 | Ile 32, Val 40, Ala 53, Met 111, Val 158, Phe 170 | Met 108, Leu 110, Asn 114, Leu 168, Thr 183 |
|  | Terpinen-4-ol | -6.1 | Val 40, Ala 53, Lys 55, Ile 86, Met 108, Leu 168, Phe 170 | Ile 32, Glu 73, Leu 106, Asp 169, Leu 172 |
|  | (4E,6E) Alloocimene | -6.5 | Ile 32, Val 40, Ala 53, Lys 55, Met 108, Val 158, Leu 168, Phe 170 | Asn 114, Thr 183 |
|  | β-Ocimene | -6.4 | Val 40, Lys 55, Ile 86, Leu 88, Leu 106, Met 108, Leu 168, Phe 170 | Glu 73, Val 107, Asp 169 |
|  | Geranyl acetate | -6.7 | Val 40, Ala 53, Lys 55, Ile 86, Leu 106, Met 108, Met 111, Val 158, Leu 168, Phe 170 | Ile 32, Leu 88, Leu 110 |
|  | β-Myrcene | -6.1 | Ile 32, Val 40, Ala 53, Lys 55, Leu 88, Leu 106, Met 108, Val 158, Leu 168, Phe 170 | Ile 86, Val 107 |
| Mice | Linalyl acetate | -6.3 | Ile 32, Val 40, Ala 53, Lys 55, Ile 86, Leu 106, Met 108, Met 111, Val 158, Leu 168 | Gly 33, Leu 88, Glu 109, Leu 110 |
|  | linalool | -5.2 | Ile 32, Val 40, Met 108, Leu 110, Met 111, Val 158, Leu 168 | Gly 33, Ala 53, Lys 55, Asn 114 |
|  | Lavandulol acetate | -6.2 | Ile 32, Val 40, Ala 53, Lys 55, Met 108, Val 158, Leu 168 | Gly 33, Glu 109, Leu 110, Met 111, Asp 112, Asn 114 |
|  | cis-β-Ocimene | -5.7 | Val 40, Ala 53, Lys 55, Ile 86, Leu 106, Met 108, Val 158, Leu 168 | Ile 32, Leu 88 |
|  | 1-Octen-3-yl-acetate | -5.4 | Val 40, Lys 55, Ile 86, Leu 88, Leu 106, Met 108, Leu 168 | Gln 37, Ala 53, Glu 73, Leu 77, Asp 169 |
|  | Terpinen-4-ol | -5.5 | Ile 32, Val 40, Met 108, Val 158, Leu 168 | Gly 33, Ala 53, Leu 110, Met 111, Asp 112, Ala 113, Asn 114, Gln 117 |
|  | (4E,6E)-Alloocimene | -6.0 | Val 40, Ala 53, Lys 55, Ile 86, Met 108, Val 158, Leu 168 | Ile 32, Leu 88, Leu 106, Met 111 |
|  | β-Ocimene | -5.9 | Val 40, Ala 53, Lys 55, Ile 86, Leu 88, Leu 106, Met 108, Val 158, Leu 168 | Ile 32 |
|  | Geranyl acetate | -6.4 | Val 40, Lys 55, Leu 77, Ile 86, Leu 106, Met 108, Leu 110, Met 111, Leu 168 | Ile 32, Ala 53, Leu 88, Glu 109, Val 158 |
|  | β-Myrcene | -5.6 | Ile 32, Val 40, Lys 55, Ile 86, Leu 88, Leu 106, Met 108, Val 158, Leu 168 | Ala 53 |


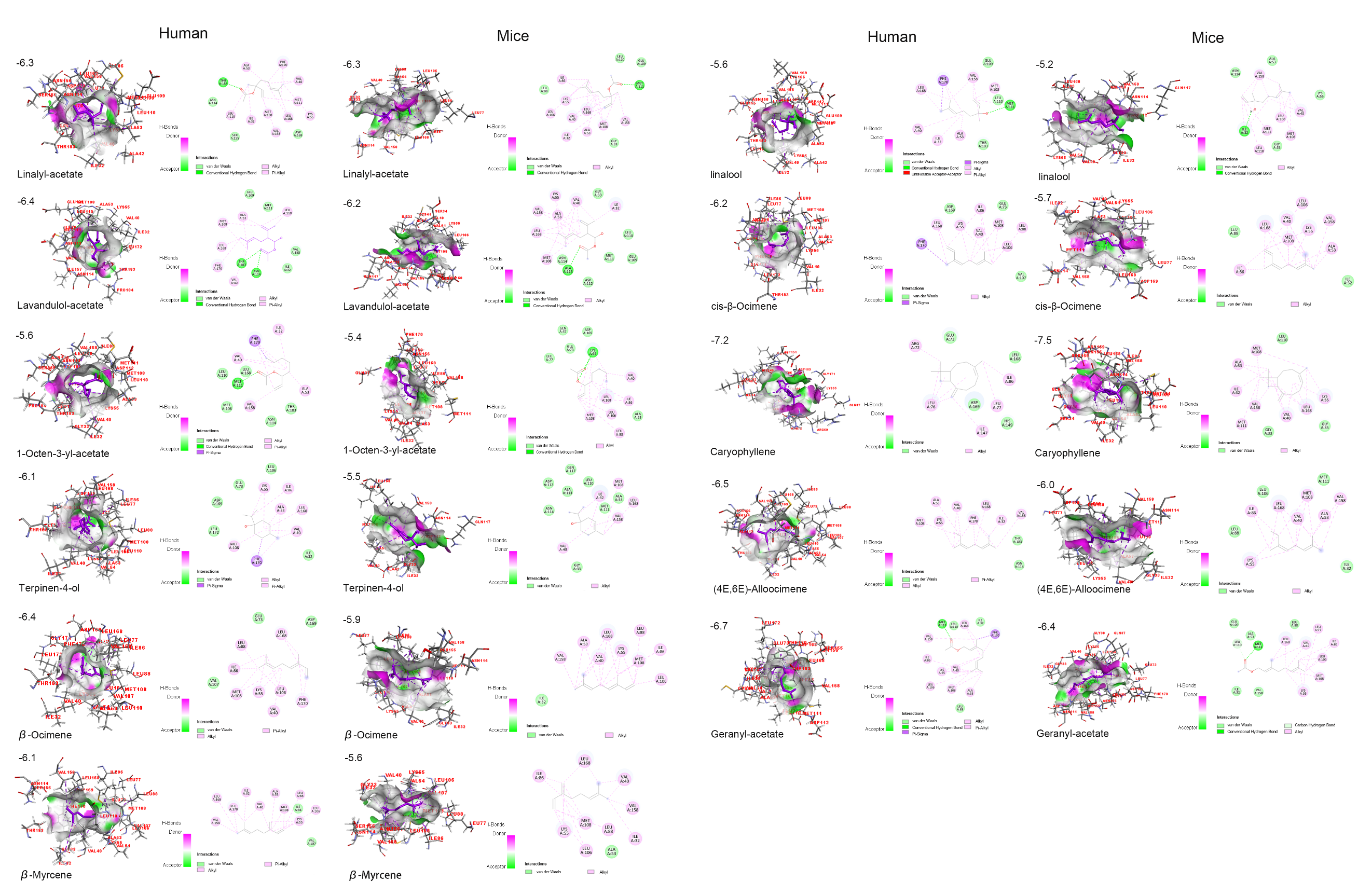


**Fig. S4:** **Molecular docking of TLEO components with Human and Mice JNK protein**

**Table S5: Molecular docking of major TLEO components with protein p50**

| **Organism** | **Compound** | **Binding Affinity**  **(kcal/mol)** | **Interaction**  **H/Alkyl/Covalent bonds** | **Van der wales bond** |
| --- | --- | --- | --- | --- |
| Human | Linalyl acetate | -5.8 | Cys 159, Leu 173, Leu 176, Arg 184, Leu 186, Leu 193, Ile 194 | Gly 166, Val 169, His 170, Asp 172, Gln 185, Gly 187, Arg 189, Glu 190 |
|  | linalool | -5.8 | Leu 173, Cys 159, Val 169, LEu 176, Arg 184, Leu 186, Leu 193, Ile 194 | His 170, Asp 172, Glu 190 |
|  | Lavandulol acetate | -6.2 | Leu 173, Gly 166, Leu 176, Arg 184, Leu 186, Leu 193 | Cys 159, Val 169, His 170, Asp 172, Gln 185, Gly 187, Arg 189, Glu 190, Ile 194, |
|  | cis-β-Ocimene | -5.8 | Cys 159, Val 169, Leu 173, Leu 176, Leu 186, Leu 193, Ile 194 | Gly 166, His 170, Asp 172, Glu 190 |
|  | 1-Octen-3-yl-acetate | -5.1 | His170, Leu176, Arg184, Cys159, Leu173, Ile 194, Leu193 | Glu 190, Gly187, Leu 186, Gln 185, Ala 174, Gly 166, Val 169, Cys 159 |
|  | Terpinen-4-ol | -6.0 | Cys 159, Val 169, His 170, Leu 173, Leu 176, Leu 193 | Asp 172, Leu 186, Glu 190, Ile 194 |
|  | (4E,6E) Alloocimene | -6.1 | Cys 159, Leu 173, Leu 176, Arg 184, Leu 186, Leu 193, Ile 194 | Val 169, His 170, Asp 172, Gln 185, Gly 187, Glu 190 |
|  | β-Ocimene | -5.8 | Cys 159, Val 169, Leu 173, Leu 176, Arg 184, Leu 186, Leu 193, Ile 194 | Ile 160, His 170, Asp 172, Gln 185, Gly 187, Glu 190 |
|  | Geranyl acetate | -6.1 | Cys 159, Val 169, Leu 176, Leu 186, Ile 194, Leu 193 | Ala 158, Asn 164, Pro 165, Gly 166, His 170, Asp 172, Leu 173, Ala 174, Tyr 175, Gln 177, Glu 190 |
|  | β-Myrcene | -5.6 | Cys 159, Val 169, Leu 173, Leu 176, Leu 193, Ile 194 | Gly 166, His 170, Asp 172, Glu 190 |
| Mice | Linalyl acetate | -5.2 | Arg 161, Tyr 163, Asn 164, Ile 94, Leu 106, Val 120, Arg 154, Ala 158 | Gln 96, Glu 157, |
|  | linalool | -5.2 | Gln 96, Leu 97, Leu 106, Val 120, Arg 154, Ala 158 | His 107, His 109, Ser 110, Leu 111, Asp 118, Gly 119, Glu 157, Arg 161, Tyr 163, Asn 164 |
|  | Lavandulol acetate | -5.6 | Leu 106, Val 120, Arg 154, Ala 158, Tyr 163 | Gln 96, Leu 97, His 107, Asp 118, Gly 119, Glu 157, Arg 161, Asn 164 |
|  | cis-β-Ocimene | -5.0 | Leu 97, Leu 106, Val 120, Arg 154, Ala 158, Tyr 163 | Gln 96, His 107, His 109, Ser 110, Leu 111, Asp 118, Gly 119, Asn 164 |
|  | 1-Octen-3-yl-acetate | -4.6 | Leu 106, Val 120, Arg 154, Ala 158, Tyr 163, Leu 168, Arg 161 | Gln 96, Leu 97, Asp 118, Gly 119, Glu 157, Asn 164 |
|  | Terpinen-4-ol | -5.1 | Glu 157, Ala 158, Val 120 | Gln 96, Leu 106, Arg 154, Arg 161, Tyr 163, Asn 164, Ieu 168 |
|  | (4E,6E)-Alloocimene | -5.1 | Leu 97, Leu 106, Val 120, Arg 154 | Gln 96, His 107, His 109, Ser 110, Leu 111, Asp 118, Gly 119, Glu 157, Ala 158, Tyr 163, Asn 164 |
|  | β-Ocimene | -4.5 | His 170, Ile 193, Ala 197 | His 105, Val 169, Ser 171, Gln 196, Gln 200, Gln 201 |
|  | Geranyl acetate | -5.2 | Arg 161, Asn 164, Ile 94, Val 120, Tyr 163, Phe 217 | Lys 92, Gln 96, Leu 106, Thr 122, Glu 157, Ala 158, Leu 168 |
|  | β-Myrcene | -4.9 | Leu 97, Leu 106, Val 120, Arg 154, Ala 158, Tyr 163, Leu 168 | Gln 96, His 107, His 109, Ser 110, Asp 118, Gly 119, Glu 157, Asn 164 |


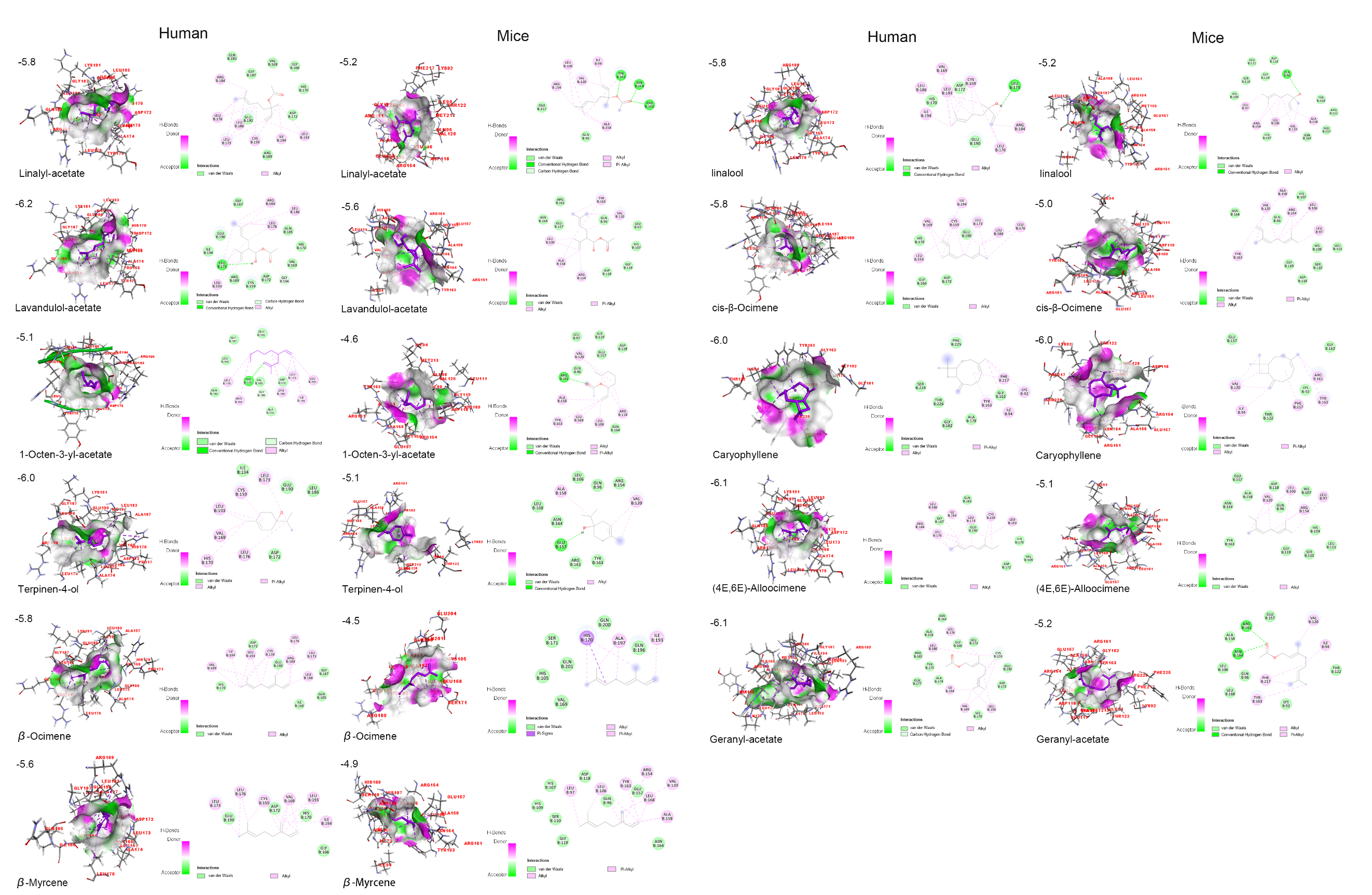


**Fig. S5: Molecular docking of TLEO components with Human and Mice p50 protein**

**Table S6: Molecular docking of major TLEO components with protein p65**

| **Organism** | **Compound** | **Binding Affinity**  **(kcal/mol)** | **Interaction**  **H/Alkyl/Covalent bonds** | **Van der wales bond** |
| --- | --- | --- | --- | --- |
| Human | Linalyl acetate | -5.0 | Lys 37, Val 121, Lys 122 | Tyr 36, Cys 38, Glu 89, Gln 119, Cys 120, Asp 125, Gln 128, Ala 129 |
|  | linalool | -5.0 | Lys 37, Val 121, Lys 122, Ala 129, Gln 132 | Glu 89, Gln 119, Cys 120, Asp 125, Gln 128, Arg 133 |
|  | Lavandulol acetate | -5.2 | Val 72, Leu 104, Cys 105, His 111, Gln 114, Asn 115 | Lys 93, Asp 94, Tyr 100, Ala 102, Glu 103, Ser 112, Phe 113 |
|  | cis-β-Ocimene | -5.0 | Tyr 36, Lys 37, Cys 38, Val 121, Lys 122 | Glu 89, Gln 119, Cys 120, Asp 125 |
|  | 1-Octen-3-yl-acetate | -4.6 | Val 72, Ala 102, Leu 104, His 111, Tyr 100, Phe 113, Gln 114 | Lys 93, Asp 94, Glu 103, Gys 105, Ser 112, Asn 115 |
|  | Terpinen-4-ol | -5.3 | Arg 35, Ala 43, Val 91 | Met 32, Gly 40, Ser 42, Gly 44, Gly 92, Gly 117, Ile 118, Gln 119 |
|  | (4E,6E) Alloocimene | -5.5 | Tyr 36, Lys 37, Cys 38, Val 121, Lys 122 | Glu 89, Gln 119, Cys 120 |
|  | β-Ocimene | -4.9 | Tyr 36, Lys 37, Cys 38, Val 121, Lys 122 | Glu 89, Gln 119, Gys 120, Asp 125 |
|  | Geranyl acetate | -5.6 | Tyr 36, Lys 37, Cys 38, Val 121, Lys 122 | Glu 89, Gln 119, Cys 120, Asp, 125, Gln 128, Ala 129, Arg 133 |
|  | β-Myrcene | -4.9 | Tyr 36, Lys 37, Cys 38, Val 121, Lys 122 | Glu 89, Gln 119, Cys 120 |
| Mice | Linalyl acetate | -4.7 | Lys 93, Tyr 100, Ala 102, Cys 105 | Val 72, Asp 94, Asp 103, Leu 104, His 111, Ser 112, Phe 113, Gln 114, Asn 115 |
|  | linalool | -4.6 | Tyr 36, Lys 37, Val 121, Ala 129, Lys 122, Asp 125 | Glu 89, Cys 120, Gln 128, Gln 132, Arg 133 |
|  | Lavandulol acetate | -5.1 | Tyr 36, Lys 37, Val 121, Lys 122, Ala 129, Arg 133 | Glu 89, Gln 119, Cys 120, Asp 125, Gln 128, Gln 132 |
|  | cis-β-Ocimene | -4.4 | Lys 37, Val 121, Lys 122, Ala 129 | Tyr 36, Glu 89, Gln 119, Cys 120, Asp 125, Gln 128 |
|  | 1-Octen-3-yl-acetate | -4.3 | Leu 126, Ile 130, Arg 149, Tyr 152 | Glu 127, Ile 134, Ile 145, Gln 148, Gly 150 |
|  | Terpinen-4-ol | -5.0 | Lys 37, Val 121, Lys 122 | Tyr 36, Glu 89, Gln 119, Cys 120, Asp 125, Gln 128, Ala 129, Arg 133 |
|  | (4E,6E)-Alloocimene | -4.8 | Lys 37, Val 121, Lys 122, Ala 129 | Tyr 36, Glu 89, Gln 119, Cys 120, Asp 125, Gln 128, Gln 132, Arg 133 |
|  | β-Ocimene | -4.6 | Tyr 36, Lys 37, Val 121, Lys 122, Ala 129 | Glu 89, Cys 120, Asp 125, Gln 128, Gln 132, Arg 133 |
|  | Geranyl acetate | -4.6 | Tyr 36, Lys 37, Val 121, Lys 122, Ala 129 | Glu 89, Cys 120, Asp 125, Gln 128, Gln 132, Arg 133 |
|  | β-Myrcene | -4.2 | Tyr 36, Lys 37, Val 121, Lys 122, Ala 129 | Glu 89, Gln 119, Cys 120, Asp 125, Gln 128 |


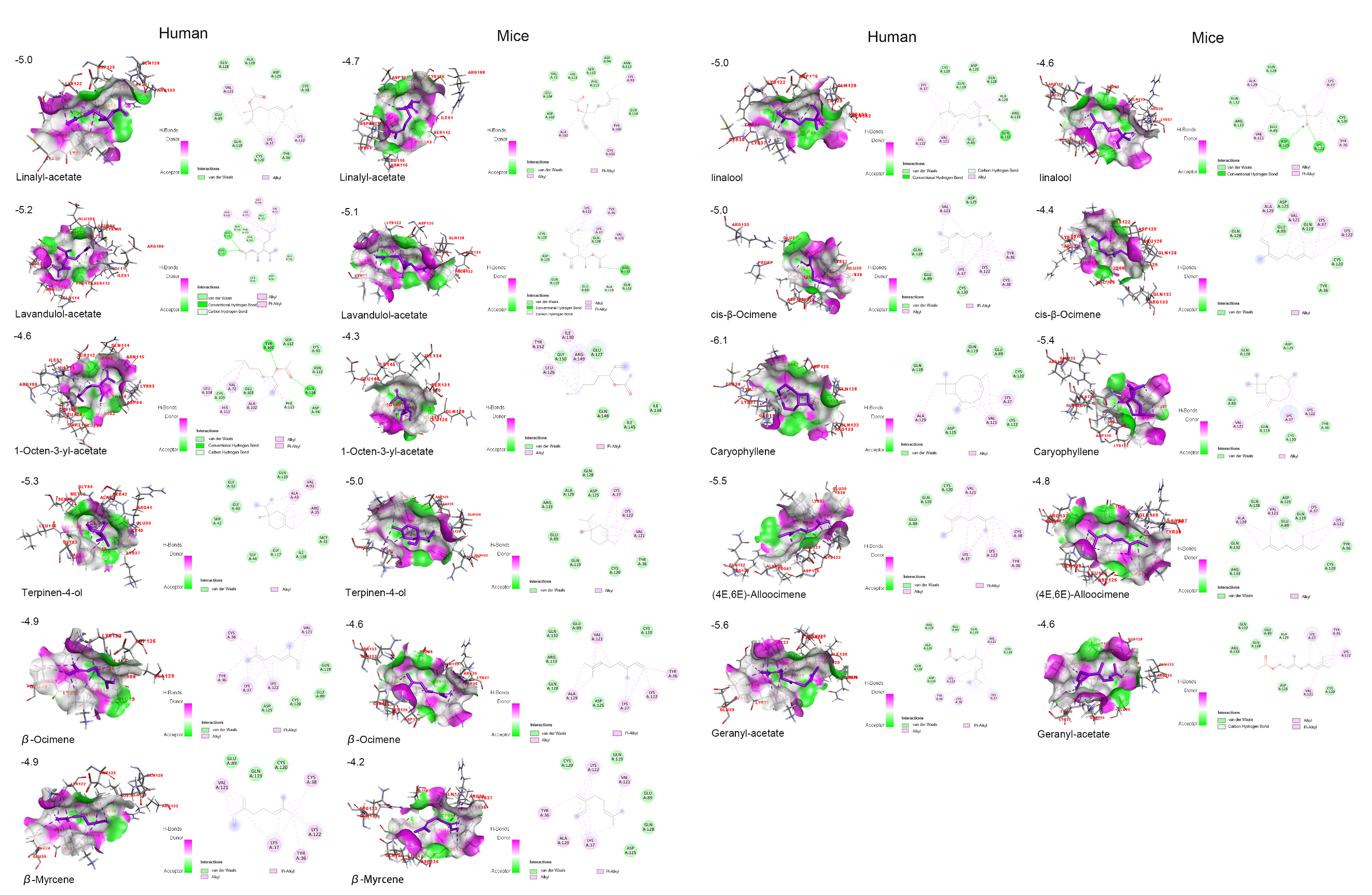


**Fig. S6:** **Molecular docking of TLEO components with Human and Mice p65 protein**

**Table S7: Molecular docking of major TLEO components with protein p38**

| **Organism** | **Compound** | **Binding Affinity**  **(kcal/mol)** | **Interaction**  **H/Alkyl/Covalent bonds** | **Van der wales bond** |
| --- | --- | --- | --- | --- |
| Human | Linalyl acetate | -5.5 | Val 38, Ala 51, Lys 53, Leu 75, Ile 84, Leu 86, Leu 104, Leu 167 | THR 106 |
|  | linalool | -5.1 | Lys 53, Val 38, Ala 51, Leu 75, Ile 84, Leu 86, Leu 104, Leu 167 | Glu 71, Val 105, Thr 106, Asp 168, Leu 171 |
|  | Lavandulol acetate | -5.8 | Lys 53, Asp 168, Tyr 35, Val 38, Leu 75, Ile 84, Ala 51, Leu 104, Leu 167 | Glu 71, Leu 86, Thr 106, Leu 171 |
|  | cis-β-Ocimene | -5.2 | Val 38, Ala 51, Lys 53, Leu 75, Ile 84, Leu 86, Leu 104, Leu 167 | THR 106 |
|  | 1-Octen-3-yl-acetate | -5.0 | Val 38, Ala 51, Lys 53, Leu 75, Ile 84, Leu 167 | Glu 71, Leu 86, Leu 104, Val 105, Asp 168, Phe 169, Leu 171 |
|  | Terpinen-4-ol | -5.6 | Arg 149, His 174, Gly 170, Leu 171 | Arg 70, Leu 74, Ile 147, His 148, Asp 150, Asp 168, Phe 169, Arg 173 |
|  | (4E,6E) Alloocimene | -5.2 | Tyr 35, Val 38, Ala 51, Lys 53, Leu 171 | Thr 106, Asp 168 |
|  | β-Ocimene | -5.1 | Val 38, Ala 51, Lys 53, Leu 75, Ile 84, Leu 86, Leu 104, Leu 167 | Thr 106, His 107 |
|  | Geranyl acetate | -5.8 | Lys 53, Tyr 35, Val 38, Ala 51, Leu 75, Ile 84, Leu 86, Leu 104, Leu 167 | Thr 106, Asp 168, Leu 171 |
|  | β-Myrcene | -5.0 | Val 38, Ala 51, Lys 53, Leu 75, Leu 86, Leu 104 | Ile 84, Thr 106, Leu 167 |
| Mice | Linalyl acetate | -5.6 | Lys 53, Leu 74, Leu 75, Ile 84, Leu 104, Thr 106, Phe 169, Leu 171 | Tyr 35, Ala 51, Glu 71, Leu 167, Asp 168 |
|  | linalool | -5.2 | Lys 53, Leu 74, Leu 75, Ile 84, Leu 104, Phe 169, Leu 171 | Tyr 35, Glu 71, Thr 106, Leu 167, Asp 168 |
|  | Lavandulol acetate | -5.7 | Val 38, Ala 51, Lys 53, Leu 75, Ile 84, Leu 167, Asp 168, Phe 169 | Tyr 35, Glu 71, Leu 74, Leu 171 |
|  | cis-β-Ocimene | -4.7 | Val 38, Ala 51, Lys 53, Ile 84, Leu 104, Met 109, Leu 167 | Tyr 35, Leu 75,  Thr 106, Asp 168 |
|  | 1-Octen-3-yl-acetate | -5.1 | Lys 53, Leu 74, Leu 75, Ile 84, Thr 106, Leu 167, Phe 169, Leu 171 | Tyr 35, Ala 51, Glu 71, Leu 104, Asp 168 |
|  | Terpinen-4-ol | -5.5 | Val 38, Ala 51, Lys 53, Leu 75, Ile 84 | Tyr 35, Leu 104, Thr 106 |
|  | (4E,6E)-Alloocimene | -5.3 | Leu 74, Leu 75, Ile 84, Leu 167, Phe 169, Leu 171 | Tyr 35, Lys 53, Glu 71, Thr 106, Met 109, Asp 168 |
|  | β-Ocimene | -4.9 | Val 38, Lys 53, Leu 75, Ile 84, Leu 104, Leu 167, Phe 169, Leu 171 | Tyr 35, Ala 51, Glu 71, Leu 74, Thr 106, Asp 168 |
|  | Geranyl acetate | -5.7 | Tyr 35, Val 38, Ala 51, Lys 53, Ile 84, Asp 168, Phe 169 | Glu 71, Leu 74, Leu 75, Thr 106, Leu 171 |
|  | β-Myrcene | -4.8 | Lys 53, Leu 75, Ile 84,Leu 104, Phe169,Leu 171 | Tyr 35, Glu 71, Leu 74, Thr 106, Leu 167, Asp 168 |


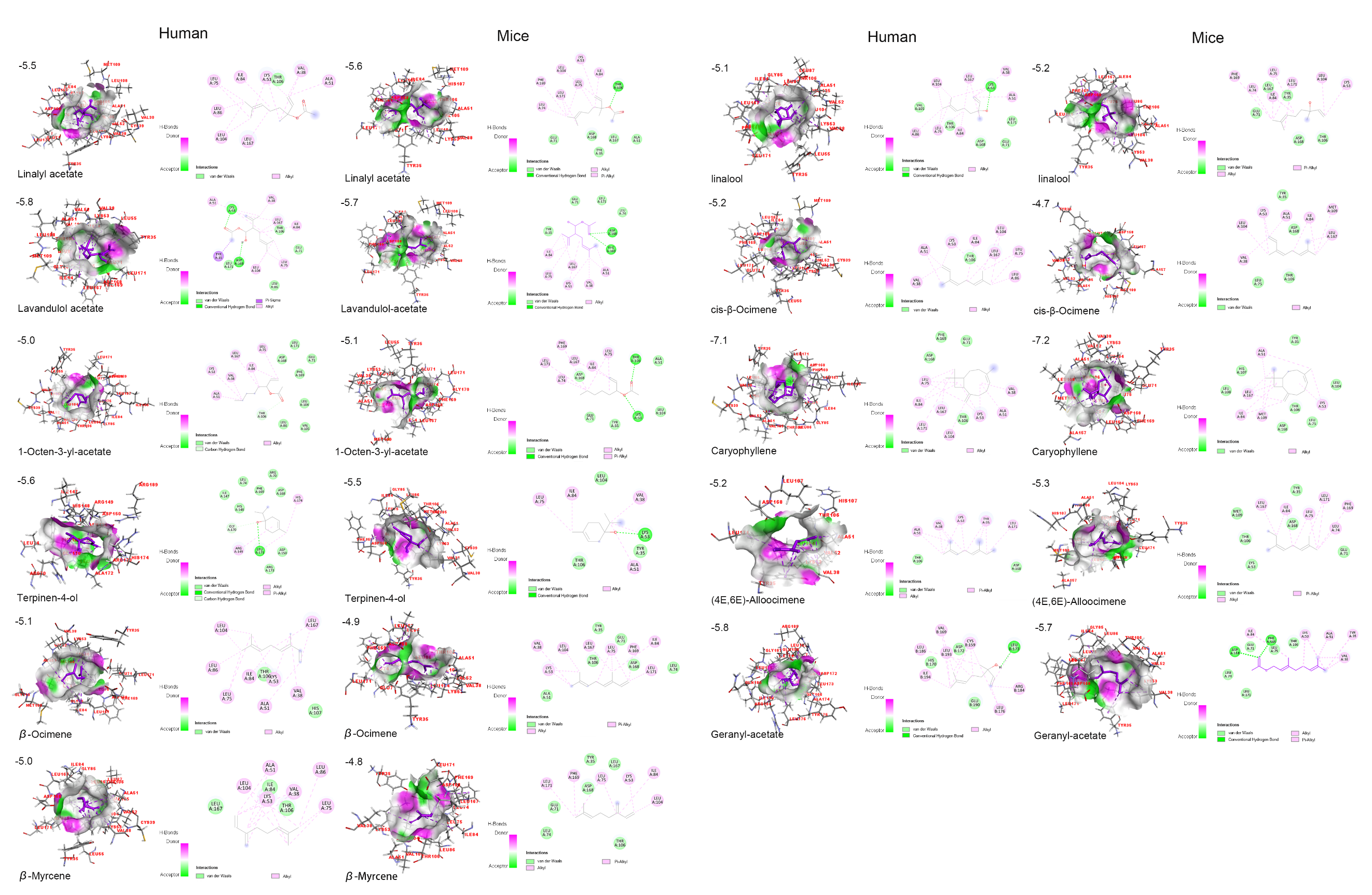


**Fig. S7:** **Molecular docking of TLEO components with Human and Mice p38 protein**


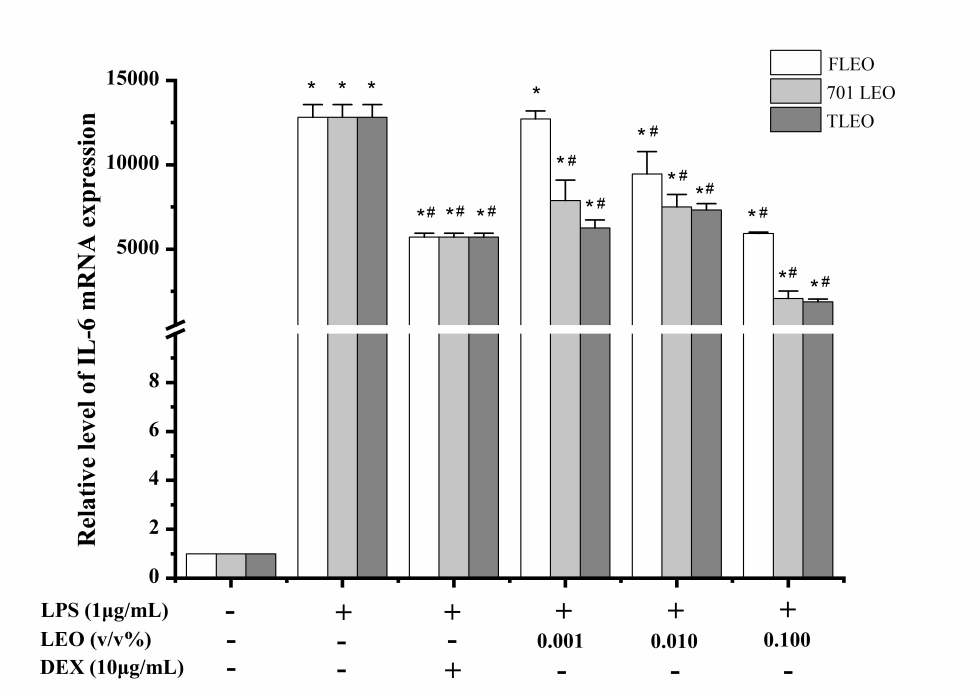


**Fig. S8: Effects of TLEO on mRNA and protein level of pro-inflammatory cytokines IL-6 by LPS-induced RAW264.7 cells. The data are the means ± S.D(n = 3). Statistical analysis was performed by one-way ANOVA with a scheffe’s test. “*” and “#” indicate significant difference (*p < 0.05*) compared with the LPS-treated group.**


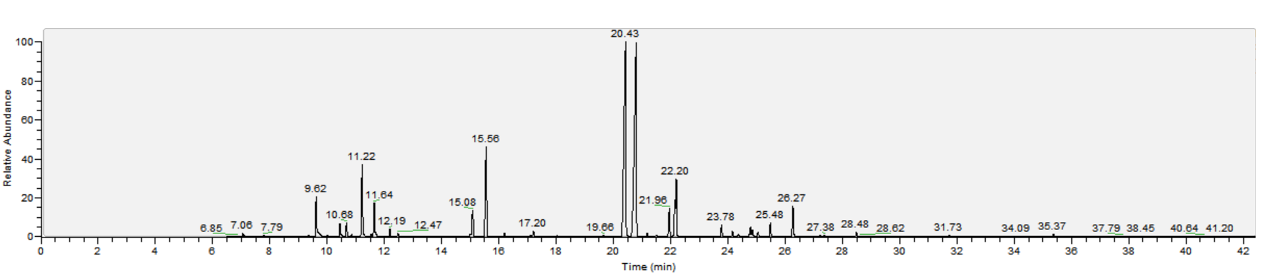


**Fig. S9: Total ion chromatography from GC-MS analysis of TLEO**
